# The Gyc76C Receptor Guanylyl Cyclase and the Foraging cGMP-Dependent Kinase Regulate Extracellular Matrix Organization and BMP Signaling in the Developing Wing of *Drosophila melanogaster*

**DOI:** 10.1371/journal.pgen.1005576

**Published:** 2015-10-06

**Authors:** Justin Schleede, Seth S. Blair

**Affiliations:** 1 Department of Zoology, University of Wisconsin, Madison, Wisconsin, United States of America; 2 Genetics Training Program, University of Wisconsin, Madison, Wisconsin, United States of America; Vanderbilt University Medical Center, UNITED STATES

## Abstract

The developing crossveins of the wing of *Drosophila melanogaster* are specified by long-range BMP signaling and are especially sensitive to loss of extracellular modulators of BMP signaling such as the Chordin homolog Short gastrulation (Sog). However, the role of the extracellular matrix in BMP signaling and Sog activity in the crossveins has been poorly explored. Using a genetic mosaic screen for mutations that disrupt BMP signaling and posterior crossvein development, we identify Gyc76C, a member of the receptor guanylyl cyclase family that includes mammalian natriuretic peptide receptors. We show that Gyc76C and the soluble cGMP-dependent kinase Foraging, likely linked by cGMP, are necessary for normal refinement and maintenance of long-range BMP signaling in the posterior crossvein. This does not occur through cell-autonomous crosstalk between cGMP and BMP signal transduction, but likely through altered extracellular activity of Sog. We identify a novel pathway leading from Gyc76C to the organization of the wing extracellular matrix by matrix metalloproteinases, and show that both the extracellular matrix and BMP signaling effects are largely mediated by changes in the activity of matrix metalloproteinases. We discuss parallels and differences between this pathway and other examples of cGMP activity in both *Drosophila melanogaster* and mammalian cells and tissues.

## Author Summary

Signaling between cells regulates many processes, including the choices cells make between different fates during development and regeneration, and misregulation of such signaling underlies many human pathologies. To understand how such signals control developmental decisions, it is necessary to elucidate both how cells regulate and respond to different levels of signaling, and how different types of signals combine and regulate each other. We have used genetic screening in the fruitfly *Drosophila melanogaster* to identify mutations that reduce or eliminate signals carried by Bone Morphogenetic Proteins (BMPs), and show that BMP signaling is sensitive Gyc76C, a peptide receptor that stimulates the production of cGMP in cells. We identify downstream intracellular effectors of this cGMP activity, but provide evidence that the effects on the BMP pathway are not mediated at the intracellular level, but rather through cGMP’s effects upon the extracellular matrix and matrix-remodeling proteinases, which in turn affects the activity of extracellular BMP-binding proteins. We discuss differences and parallels with other examples of cGMP activity in *Drosophila melanogaster* and mammals.

## Introduction

The vein cells that develop from the ectodermal epithelia of the *Drosophila melanogaster* wing are positioned, elaborated and maintained by a series of well-characterized intercellular signaling pathways. The wing is easily visualized, and specific mutant vein phenotypes have been linked to changes in specific signals, making the wing an ideal tissue for examining signaling mechanisms, for identifying intracellular and extracellular crosstalk between different pathways, and for isolating new pathway components [1–3].

**Fig 1:**
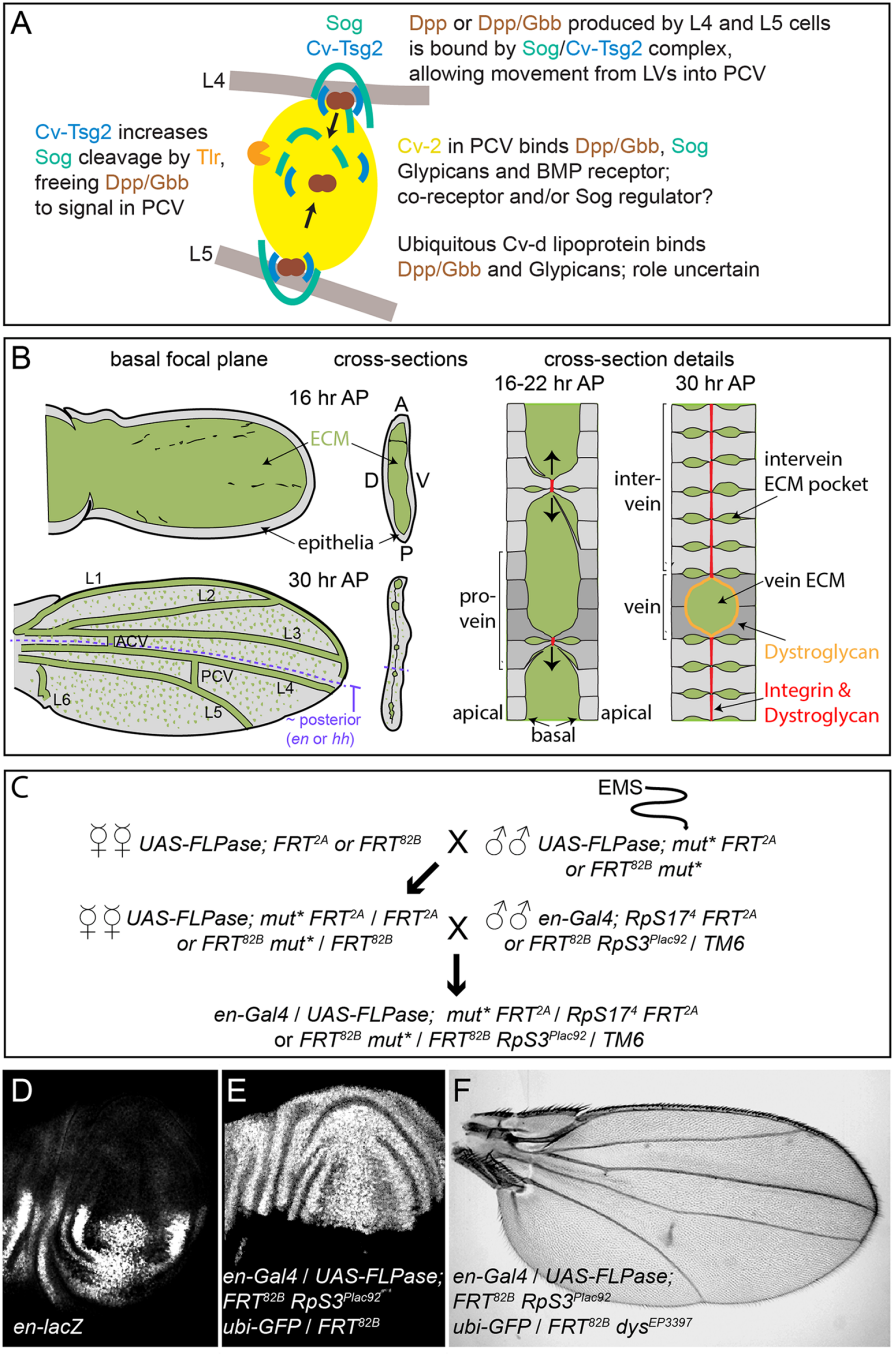
PCV development and the genetic screen. (A) Model of signaling in the PCV. BMPs (Dpp and Gbb) secreted by adjacent LV cells bind to the Sog/Cv-Tsg2 complex, allowing movement into the PCV region. Cv-Tsg2 helps stimulate cleavage of Sog by the Tlr protease, freeing BMPs for signaling. Cv-2, largely bound to cells by glypicans, also binds BMPs, BMP receptors and Sog, locally transferring BMPs from Sog to the receptor complex. Cv-d also binds glypicans and BMPs and increases signaling by an unknown mechanism. (B) Diagram of vein and ECM development during the period of PCV formation in low magnification and high magnification cross-sections. As the dorsal and ventral epithelia reattach, the basal ECM of the early wing is remodeled, coming to lie in the vein channels and in scattered basolateral pockets between cells. Integrins and Dystroglycan concentrate at sites of basal-to-basal cell adhesion (both) and lining the veins (Dystroglycan). (C) Crossing scheme used to generate large homozygous mutant clones throughout the posterior compartment of the developing wing in heterozygous flies. *UAS-FLPase; FRT* ^*2A*^ (3L) or *UAS-FLPase; FRT* ^*82B*^ (3R) males were fed EMS and backcrossed to virgin females of the same genotype. mut* represents EMS-mutagenized chromosome. Virgin female F1 progeny were then crossed to *en-Gal4; FRT* ^*82B*^, *RpS3* ^*Plac92*^ *Ubi-GFP / TM6* or *en-Gal4*; *hs-GFP RpS17* ^*4*^ *FRT2A /TM6* males. Approximately 50 females were used for each of the first two crosses. (D) *en* expression in posterior of late third wing discs, shown using the *en-lacZ* enhancer trap. (E) Large posterior homozygous clones, marked by the absence of GFP (white), induced in late third instar wing disc using *en-Gal4*/*UAS-FLPase*; *FRT* ^*82B*^ */FRT* ^*82B*^ *RpS3* ^*Plac92*^ *ubi-GFP*. (F) Test of the mosaic method using *en-Gal4*/*UAS-FLPase; FRT* ^*82B*^ *RpS3* ^*Plac92*^ *ubi-GFP*/*FRT* ^*82B*^ *dystrophin* ^*EP3397*^; the adult wing shows the “detached” crossvein phenotype typical of *dystrophi*n (*dys*) loss.

We and others have been using one venation defect, the loss of the posterior crossvein (PCV), to identify and characterize participants in Bone Morphogenetic Protein (BMP) signaling. The PCV is formed during the end of the first day of pupal wing development, well after the formation of the longitudinal veins (LVs, numbered L1-L6) (Fig 1B), and requires localized BMP signaling in the PCV region between L4 and L5 [4]. Many of the homozygous viable crossveinless mutants identified in early genetic screens have now been shown to disrupt direct regulators of BMP signaling, especially those that bind BMPs and regulate BMP movement and activity in the extracellular space [5, 6]. The PCV is especially sensitive to loss of these regulators because of the long range over which signaling must take place, and the role many of these BMP regulators play in the assembly or disassembly of a BMP-carrying “shuttle”.

As summarized in Fig 1A, the BMP Decapentaplegic (Dpp) is secreted by the pupal LVs, possibly as a heterodimer with the BMP Glass bottom boat (Gbb). This stimulates autocrine and short-range BMP signaling in the LVs that is relatively insensitive to extracellular BMP regulators. However, Dpp and Gbb also signal over a long range by moving into the intervein tissues where the PCV forms [7–9]. In order for this to occur, the secreted BMPs must bind the *D*. *melanogaster* Chordin homolog Short gastrulation (Sog) and the Twisted gastrulation family member Crossveinless (Cv, termed here Cv-Tsg2 to avoid confusion with other “Cv” gene names). The Sog/Cv-Tsg2 complex facilitates the movement of BMPs from the LVs through the extracellular space, likely by protecting BMPs from binding to cell bound molecules such as their receptors [8–11]. In order to stimulate signaling in the PCV, BMPs must also be freed from the complex. The Tolloid-related protease (Tlr, also known as Tolkin) cleaves Sog, lowering its affinity for BMPs, and Tsg family proteins help stimulate this cleavage [12, 13]. Signaling is further aided in the PCV region by a positive feedback loop, as BMP signaling increases localized expression of the BMP-binding protein Crossveinless 2 (Cv-2, recently renamed BMPER in vertebrates). Cv-2 also binds Sog [14](Olsen, Halbisen, Li and Blair, in preparation), cell surface glypicans and the BMP receptor complex, and likely acts as a co-receptor and a transfer protein that frees BMPs from Sog [8, 15, 16]. The lipoprotein Crossveinless-d (Cv-d) also binds BMPs and glypicans and helps signaling by an unknown mechanism [17].

PCV development takes place in a complex and changing extracellular environment, but while there is some evidence that PCV-specific BMP signaling can be influenced by changes in tissue morphology [18] or loss of the cell-bound glypican heparan sulfate proteoglycans [17], other aspects of the environment have not been greatly investigated. During the initial stages of BMP signaling in the PCV, at 15–18 hours after pupariation (AP), the dorsal and ventral wing epithelia form a sack that retains only a few dorsal to ventral connections from earlier stages; the inner, basal side of the sack is filled with extracellular matrix (ECM) proteins, both diffusely and in laminar aggregates (Fig 1B) [19–24]. As BMP signaling in the PCV is maintained and refined, from 18–30 hours AP, increasing numbers of dorsal and ventral epithelial cells adhere, basal to basal, flattening the sack. The veins form as ECM-filled channels between the two epithelia, while in intervein regions scattered pockets of ECM are retained basolaterally between the cells within each epithelium; a small amount of ECM is also retained at the sites of basal-to-basal contact. This changing ECM environment could potentially alter BMP movement, assembly of BMP-containing complexes, and signal reception, as has been demonstrated in other developmental contexts in Drosophila [25–30].

We will here demonstrate the strong influence of the pupal ECM on PCV-specific long-range BMP signaling, through the identification of a previously unknown ECM-regulating pathway in the wing. In a screen we conducted for novel crossveinless mutations on the third chromosome, we found a mutation in the *guanylyl cyclase at 76C* (*gyc76C*) locus, which encodes one of five transmembrane, receptor class guanylyl cyclases in *D*. *melanogaster* [31–33]. Gyc76C has been previously characterized for its role in Semaphorin-mediated axon guidance; Malpighian tubule physiology, and the development of embryonic muscles and salivary glands [34–39]. Like the similar mammalian natriuretic peptide receptors NPR1 and NPR2 [40], the guanylyl cyclase activity of Gyc76C is likely regulated by secreted peptides [35], and can act via a variety of downstream cGMP sensors.

Our evidence suggests that Gyc76C influences BMP signaling in the pupal wing by changing the activity of the cGMP-dependent kinase Foraging (For; also known as Dg2 or Pkg24A) [41], also a novel role for this kinase. But rather than controlling BMP signal transduction in a cell-autonomous manner, we will provide evidence that Gyc76C and Foraging regulate BMP signaling non-autonomously by dramatically altering the wing ECM during the period of BMP signaling in the PCV. This effect is largely mediated by changing the levels and activity of matrix metalloproteinases (Mmps), especially Drosophila Mmp2. Genetic interactions suggest that the ECM alterations affect the extracellular mobility and activity of the BMP-binding protein Sog.

This provides the first demonstration of Gyc76C and For activity in the developing wing, and the first evidence these proteins can act by affecting Mmp activity. Moreover, our demonstration of in vivo link from a guanylyl cyclase to Mmps and the ECM, and from there to long-range BMP signaling, may have parallels with findings in mammalian cells and tissues. NPR and NO-mediated changes in cGMP activity can on the one hand change matrix metalloproteinase expression secretion and activity (e.g. [42–47]), and on the other change in BMP and TGFβ signaling [48–52]; we will discuss these below.

## Results

### Genetic screen for new crossveinless mutations

As many critical regulators of BMP signaling are likely to be required at earlier stages of *D*. *melanogaster* development, we screened for novel BMP regulators in the PCV by creating large mitotic recombinant clones homozygous for mutagenized third chromosomes in the posterior, PCV territory of the developing wing. We utilized posteriorly-expressed *engrailed* (*en*)*-Gal4* and *UAS- FLPase* to induce mitotic recombination between mutagenized FRT-bearing chromosome [53, 54]; the non-mutagenized FRT chromosomes carried *Minute* (*M*) mutations that dominantly slow the rate of cell divisions (*RpS17* for the 3R chromosome arm and *RpS3* for 3L), allowing the homozygous mutagenized clones to divide more quickly and outcompete their *M* ^*+*^ */M* ^*-*^ neighbors [55]. In *en-Gal4 / UAS-FLP; FRT* ^*82B*^ *RpS3* ^*Plac92*^ *ubi-GFP / FRT* ^*82B*^ wing discs, homozygous wild type (*M* ^*+*^) clones, identified by the absence of GFP, fill almost the entire posterior (Fig 1E). Inducing clones homozygous for a recessive mutation in *dystrophin* (*dys*) using *en-Gal4 / UAS-FLP; FRT* ^*82B*^ *RpS3* ^*Plac92*^ *ubi-GFP / FRT* ^*82B*^ *dys* ^*EP3397*^ reliably generated adults with a partial, “detached” PCV phenotype (Fig 1F), similar to that caused by loss of *dys* function in the entire wing [56].

**Fig 2:**
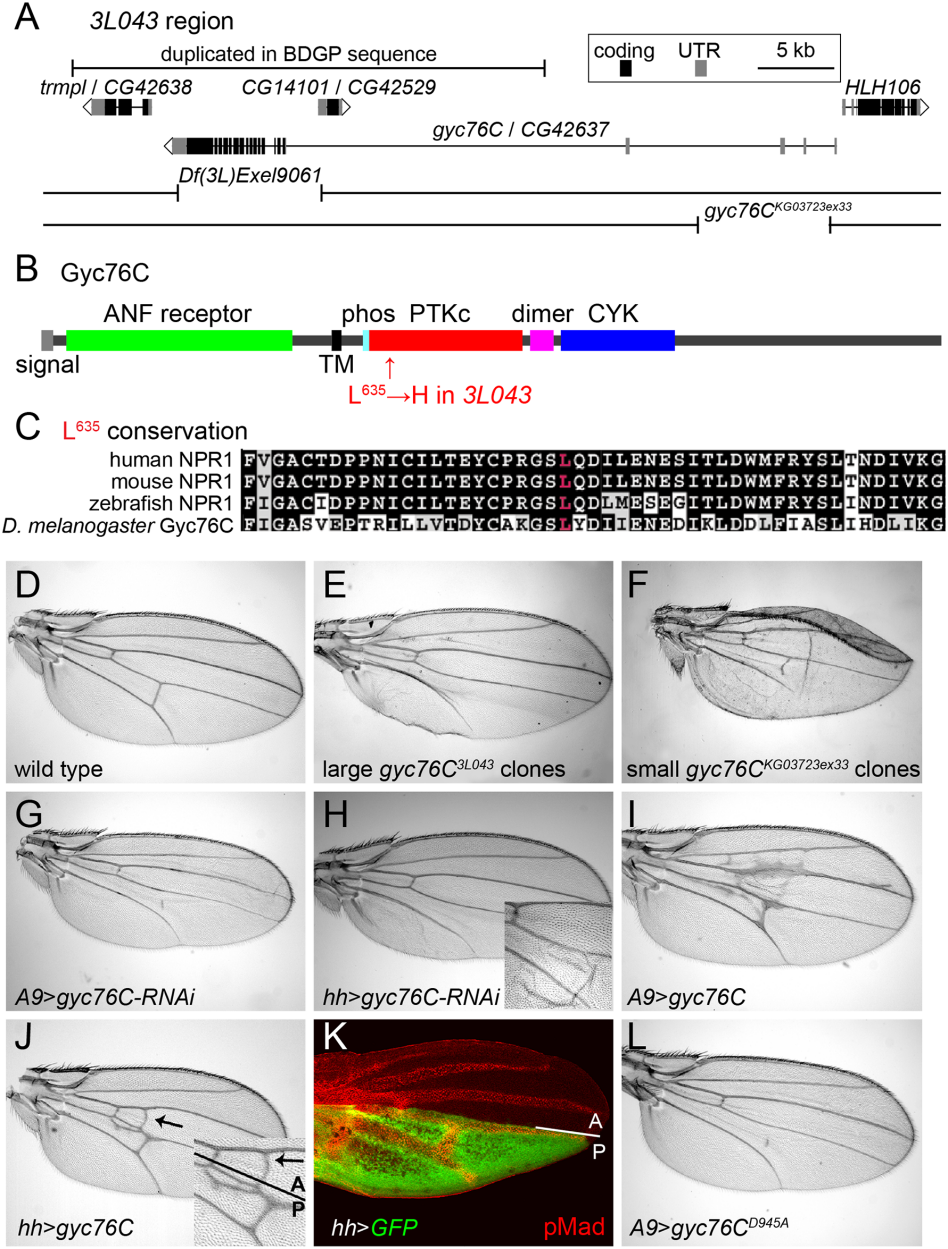
Mapping of *gyc76C* ^*3L043*^ and characterization of *gyc76C* vein phenotypes. (A) *3L043* genomic region. *3L043* failed to complement the deficiencies *Df(3L)Exel9061* and *gyc76C* ^*KG03723ex33*^. A bar marks a genomic region duplicated in the subset of the *iso-1* strain used to generate the Berkeley *Drosophila* Genome Project (BDGP) genomic sequence, but unlikely to be duplicated in the mutagenized strain [57] (S2 Fig). (B) Domain structure of the Gyc76C protein, with an N-terminal, extracellular ANF receptor domain, a transmembrane (TM) domain, a putative phosphorylation site (phos), a kinase “dead” PTKc domain, a putative dimerization domain (dimer), and a C-terminal CYC domain. The L^635^H mutation of *gyc76C* ^*3L043*^ is in the N-terminal region of the PTKc domain. (C) Conservation of Gyc76C’s L^635^ (red) in vertebrate NPR1s. (D) Wild type adult wing. (E) Crossveinless phenotype resulting from large posterior homozygous *gyc76C* ^*3L043*^ mutant clones in *en-Gal4/UAS-FLP; gyc76C* ^*3L043*^ *FRT* ^*2A*^/*hs-GFP RpS17* ^*4*^ *FRT* ^*2A*^ fly. (F) Crossvein and L5 disruption resulting from smaller *gyc76C* ^*KG0372ex33*^ clones in *hs-FLP; gyc76C* ^*KG0372ex33*^ *FRT* ^*2A*^ */hs-GFP RpS17* ^*4*^ *FRT* ^*2A*^ fly. (G,H) PCV disruption caused by *A9-Gal4*-driven (G) or *hh-Gal4*-driven (H) expression of *UAS-gyc76C-RNAi* (VDRC 6552). Detail in H shows wing with blistering phenotype typical of stronger Gal4 expression at 28°C. (I,J) Ectopic venation caused by *A9-Gal4*-driven (I) or *hh-Gal4*-driven (J) expression of *UAS-gyc76C*. Detail in (J) shows that the ectopic venation induced by *hh-Gal4* extends anterior to the A/P compartment boundary. (K) Pupal *hh-Gal4 UAS-GFP* wing at 24 hours AP showing GFP expression (green) posterior to the A/P compartment boundary and its position relative to the veins, marked with anti-pMad (red). (L) PCV disruption and lack of ectopic venation caused by *A9-Gal4*-driven expression of the cyclase dead *UAS-myc-gyc76C* ^*D945A*^.

We screened 14,500 F2 adults; the 9 independent mutant chromosomes we found that reliably disrupted the PCV in large posterior clones were recessive and homozygous lethal. One 3L mutant chromosome gave crossveinless phenotypes over *vvl* ^*M638*^ and *vvl* ^*sep*^ and three 3R mutant chromosomes were lethal over *dys* ^*EP3397*^. The remaining 5 (Fig 2) (S1 Fig) complemented these and other candidates known to be required for PCV or vein formation, and complemented each other. In addition, one viable mutant found originally in an F2 male was not caused by third chromosome recombinant clones, but mapped to the X chromosome, and is allelic to *ade5* as will be discussed below.

### *3L043* mutates *guanylyl cyclase at 76C*

Large posterior *3L043* clones result in the complete loss of the PCV in adult wings (Fig 2E). Deletion mapping placed the lethality under *Df(3L)Exel9061*, a molecularly-defined deletion [58] that removes part of *CG14101* and the coding exons of *gyc76C* (Fig 2A). *3L043* was lethal over *gyc76C* ^*KG0373ex33*^, a homozygous lethal 8kb genomic deletion that leaves *CG14101* intact but removes part of the 5’ UTR of *gyc76C* [34] (Fig 2A). Sequencing *3L043* DNA identified an A to T transversion within exon 15 of *gyc76C*, resulting in the missense mutation L635H (Fig 2B and 2C).

Loss of *gyc76C* function mimicked the *3L043* phenotype. Flies carrying small homozygous *gyc76C* ^*KG0373ex33*^ clones, generated using *heat shock promoter 70 (hs)*-driven *FLPase*, often had wings with disrupted PCVs (Fig 2F). Flies carrying larger posterior *gyc76C* ^*KG0373ex33*^ clones, generated using *en-Gal4 UAS-FLPase* and the *Minute* technique, did not survive to adulthood, but driving expression of a *UAS-RNAi* constructs directed against *gyc76C* (*UAS-gyc76C-RNAi*, VDRC stocks 3057 and 6552), with either the general disc driver *A9-Gal4* or the posterior wing driver *hh-Gal4*, caused partial or complete loss of the PCV (Fig 2G and 2H). *gyc76C* ^*KG0373ex33*^ clones and *hh-Gal4*-driven expression of *UAS-gyc76C-RNAi* also caused occasional morphological defects and wing blistering not seen with large posterior *gyc76C* ^*3L043*^ clones (Fig 2G and 2H), suggesting that the *gyc76C* ^*3L043*^ allele is hypomorphic.

Gyc76C shares the intracellular domain structure of vertebrate NPRs (Fig 2B), including an intracellular protein tyrosine-like kinase (PTKc) domain, a putative coiled-coil dimerization domain (“dimer” in Fig 2B), and a guanylyl cyclase catalytic (CYC) domain [59]. The L635H missense mutation in *gyc76C* ^*3L043*^ alters a residue within the PTKc domain that is conserved in vertebrate NPRs (Fig 2C). However, the Gyc76C PTKc domain, like that of the NPRs, lacks a glutamate that is required to catalyze phosphate transfer and thus is likely kinase dead [60]; this domain is thought to regulate the activity of the guanylyl cyclase domain [39, 59].

### Gyc76C likely acts via cGMP and the cGMP-dependent kinase For

While receptor guanylyl cyclases can increase cGMP levels, vertebrate NPR-A can also act independently of cGMP by directly binding to and altering the activity of TRPC3/C6 Ca^2+^ channels [61]. Two lines of evidence strongly suggest, however, that Gyc76C is acting in the wing via the production of cGMP. First, we compared the effects of overexpressing wild type and cyclase-dead versions of the Gyc76C. Overexpression of wild type *gyc76C* in the wing induces ectopic venation (Fig 2I and 2J). In other contexts expressing a form of Gyc76C carrying a D945A mutation within its CYC domain has a dominant negative effect on endogenous Gyc76C cyclase activity, likely through the formation of non-functional homodimers as occurs with a similar mutation in NPR [34, 62]. Overexpression of *UAS-myc-gyc76C* ^*D945A*^ using *hh-Gal4* or *A9-Gal* not only failed to induce ectopic venation, but caused crossveinless phenotypes (Fig 2L). This is in marked contrast to the equivalent mutation in NPR-A, which retained its ability to alter TRPC3/C6 channel activity [61].

Second, cGMP can act by stimulating cGMP-dependent protein kinases (PRKGs), and loss of one of these mimicked the *gyc76C* mutant phenotype. *D*. *melanogaster* has three PRKGs: Pkg21D (also known as Dg1), CG4839, and For (also known as Dg2) [41, 63, 64]. RNAi-mediated knockdown of Pkg21D mimics the loss of Gyc76C in Malpighian tubule function, axon guidance and embryonic muscle and salivary gland formation [35, 37–39]. However, *en-Gal4*-driven or *A9-Gal4*-driven expression of *UAS-pkg21D-RNAi* (VDRC 34594 or 34595) did not disrupt PCV formation. *CG4839* ^*MB10509*^ flies have a Minos transposable element inserted into one of the gene’s coding exons but are homozygous viable with normal wing venation.

**Fig 3:**
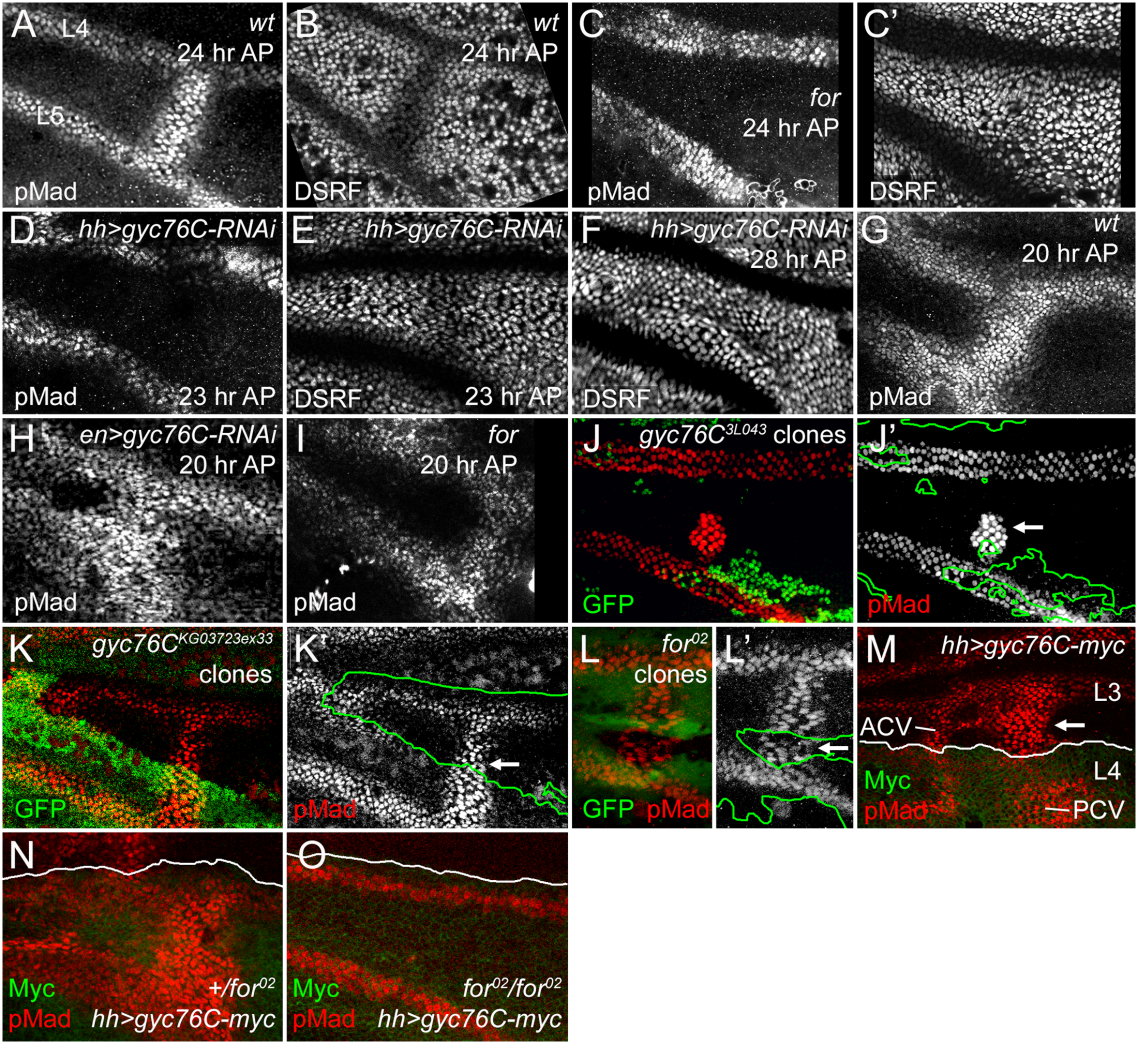
For and Gyc76C are required for the refinement and maintenance of BMP signaling. (A,B) PCV regions of 24 hour AP wild type (wt) wings showing anti-pMad staining (A) and suppression of anti-DSRF expression (B) in veins. (C,C’) 24 hour AP *for* ^*02*^ homozygote wing showing loss of loss of pMad (C) and failure to suppress DSRF (C’) in PCV. (D,D) 23 hour AP *hh-Gal4 UAS-gyc76C-RNAi* wings showing loss of pMad (D) but still slight suppression of DSRF (E) in PCV. (F) 28 hour AP *hh-Gal4 UAS-gyc76C-RNAi* wing showing failure to suppress DSRF in PCV. (G) Anti-pMad staining in 20 hour AP wild type wing. (H) 20 hour AP *en-Gal4 UAS-gyc76C-RNAi* wing showing abnormally broad anti-pMad in PCV and LVs. (I) 20 hour AP *for* ^*02*^ homozygous wing showing pMad in PCV. (J-K’) Anti-pMad staining (red, white) in homozygous *gyc76C* ^*3L043*^ (J,J’) or *gyc76C* ^*KG0372ex33*^ (K,K’) clones (identified by absence of green GFP) in 28 hour AP *hs-FLP/+; gyc76C FRT* ^*2A*^ */hs-GFP RpS17* ^*4*^ *FRT* ^*2A*^ wings. Individual cells in PCV retain high levels of pMad (arrows), similar to levels in neighboring wild type PCV or LV cells. (L,L’) Normal anti-pMad staining (red, white) in PCV cells of homozygous *for* ^*02*^ clone (identified by absence of green RFP) in *hsFlp*; *for* ^*02*^ *FRT* ^40A^/*ubi-RFP FRT* ^*40A*^ 26 hour AP wing. (M) Anti-pMad (red) and anti-Myc (green) staining in a 24 hour AP *UAS-myc-gyc76C*/*+*; *hh-Gal4*/*+* wing. Arrow indicates ectopic pMad between L3 and L4 anterior to the PCV, outside the region of *hh-Gal4*-driven expression of Myc-Gyc76C. (N,O) Comparison of anti-pMad (red) staining in 25 hour +/*for* ^*02*^ and *for* ^*02*^/*for* ^*02*^ wings with *hh-Gal4 UAS-gyc76C-myc*/+ (anti-Myc, green). Ectopic pMad observed in *for* heterozygote (N) is lost in *for* homozygote (O).

By contrast, PCVs were lost from the wings of pupae homozygous for the adult lethal *for* ^*k04703*^ and *for* ^*02*^ alleles. PCVs can normally be visualized from 16 to 32 hours AP using antisera specific to the C-terminal phosphorylated form of the BMP receptor-activated Smad, Mothers against Dpp (anti-pMad), and after 22–24 hours AP by reduced expression of *D*. *melanogaster* Serum Response Factor transcription factor (DSRF, also known as Blistered) (Fig 3A and 3B) [4]. 24 hour AP *for* homozygotes lacked anti-pMad staining and DSRF downregulation in the PCV (Fig 3C and 3C’). Removal of *for* also blocks the effects of Gyc76C overexpression: *hh-gal4*-induced overexpression of *gyc76C* induced ectopic anti-pMad staining in pupal wild type or *for* ^*02*^/+ wings, especially in a central region of the wing near the normal crossveins (Fig 3M and 3N), but did not do so in *for* ^*02*^ homozygotes (8/8 cases) (Fig 3O). We will show below that mutation of *for* not only mimics the effects of *gyc76C* knockdown on BMP signaling, but also its effects on the ECM, strongly supporting the involvement of Gyc76C and the PRKG For in a common pathway linked by cGMP.

A role for cGMP in crossvein-specific BMP signaling provides an explanation for the crossveinless wings produced by defects in purine synthesis, a part of the “purine syndrome” [65–67]. As noted above, a homozygous viable X chromosome crossveinless mutation found in our screen (*X1*) maps to and is allelic to the *ade5* gene (S3 Fig), which encodes an enzyme with 5-aminoimidazole ribonucleotide carboxylase and 4-[(N-succinylamino)carbonyl]-5-aminoimidazole ribonucleotide synthetase activities, the sixth and seventh steps in purine synthesis [68]. The pupal crossvein defects in *ade5* ^*1*^ wings were similar to, although milder than, those caused by *for* mutations (S3E and S3F Fig).

We also attempted to manipulate cGMP in the wing using the *D*. *melanogaster* cGMP phosphodiesterase PDE6, which can reduce levels of cGMP (and cAMP in one assay) after extraction from S2 cells [69]; overexpression of PDE6 causes a 25% reduction of cGMP levels in Malpighian tubules [70]. However, *UAS-PDE6-RNAi* driven with *hh-Gal4* or *en-Gal4 UAS-dcr2 UAS-PDE6-RNAi* only rarely produced the ectopic venation expected from increased cGMP, and overexpression of wild type *UAS-PDE6* or a mutated form lacking a prenylation site that alters its subcellular localization [71], did not produce crossveinless wings with either *hh-Gal4*, *en-Gal4*, or *MS1096-Gal4*. While the cGMP reductions caused by PDE might be expected to block PRKG activity, even ubiquitous PDE5/6 overexpression with *actin promoter*-driven *Gal4* failed to reproduce the lethality of *for* or *Pkg21D* mutants, and adults appeared normal. PDE activity can be regulated at several levels, and cGMP, PDE and PRKG activities can depend greatly on subcellular localization [72, 73]. Given our other experimental support for cGMP’s role in the *gyc76C* and *for* phenotypes, we think it likely that PDE6 does not cause a large enough cGMP change, in the correct subcellular compartment, to greatly affect For activity.

We next investigated the role of the only known ligand for Gyc76C, but found it plays only a weak role in the wing. The VQQ neuropeptide, one of several produced from the Nplp1 peptide precursor protein, can stimulate Gyc76C-dependent cGMP production in S2 cells and Malpighian tubules, although the effects of its removal have not been tested [35]. *Nplp1* ^*EY11089*^ is a P element insertion that introduces stop codons into the first coding exon of *Nplp1*, 3’ to the signal peptide-coding region needed for secretion, but 5’ to the peptide coding region (S4A Fig). But while this mutation blocked Nplp1 peptide production in the CNS (S4B and S4C Fig), it failed to reproduce the lethality of strong *gyc76C* mutants, and caused only occasional ectopic branching from the PCV rather than its loss (S4D and S4E Fig). Thus, either there are redundant Gyc76C-stimulating peptides, or Gyc76C has significant activity in the absence of peptide binding. Plexin A-mediated Semaphorin signaling can affect Gyc76C activity in embryonic axons and in vitro [34, 39], but we have reduced Plexin A signaling in the wing and found no effects on PCV development (*hh-Gal4 UAS-Plexin A-RNAi*).

### Gyc76C and For act non-autonomously to refine and maintain normal BMP signaling in the pupal wing

The LVs are specified early in wing development by localized EGF-receptor-mediated MAPK activity, well prior to the appearance of the crossveins, but begin to express the BMP Dpp during early pupal stages [1, 8, 74]. Anti-pMad provides a measure of BMP signaling immediately downstream of receptor activation; anti-pMad staining appears around both the LVs and the PCV at 15 hours AP; by 18–20 hours the PCV always forms a continuous, gap-less line of pMad staining between L4 and L5, despite the PCV not expressing Dpp or requiring EGF receptor-mediated MAPK activity until after 24 hours AP [4, 8]. As in *for* mutants, knockdown of *gyc76C* using *hh-Gal4*-driven or *en-Gal4*-driven expression of *UAS-gyc76C-RNAi*, always blocked or created large gaps in anti-pMad staining in the 23–24 hour AP PCV, as shown by comparing pMad levels with those in the adjacent LVs (Fig 3D and S5D Fig). This was accompanied by loss of DSRF downregulation in the PCV between 24 and 28 hours AP, slightly later than the equivalent effect in *for* mutants (Fig 3C’, 3E and 3F, S5D’ Fig).

However, BMP signaling was still initiated in the PCVs of *for* mutant or *gyc76C* knockdown wings, and visible at 20–22 hours AP (Fig 3H and 3I, S5C Fig). In *for* mutants signaling was often reduced at stages prior to formation of a vein lumen in the PCV region (S6 Fig). The early BMP signaling was more robust after *gyc76C* knockdown than in *for* mutants; in fact, *en-Gal4*-driven knockdown of *gyc76C* often led to broader anti-pMad staining than in wild type wings at 20 hours AP, in both the PCV and the LVs (Fig 3G and 3H). The broadening of the PCV and adjacent LVs was also apparent in the dorsal epithelium after driving dorsal-specific knockdown using *ap-Gal4 UAS-gyc76C-RNAi* (S5A Fig) and the width of L5 in adult *hh-Gal4 UAS-gyc76C-RNAi* wings was also significantly greater than in control *hh-Gal4* wings (S7A–S7E Fig).

Thus, the effects on BMP signaling were complex: Gyc76C suppressed and refined BMP signaling around the LVs and the early PCV, but Gyc76C and For maintained BMP signaling in the older PCV. This is difficult to reconcile with an intracellular, “cell-autonomous” effect of Gyc76C and For on BMP signal transduction, which would be expected to lower pMad levels in all the vein cells. Instead, the effect is quite reminiscent of extracellular alterations in long-range BMP signaling: reducing the BMP shuttling mediated by extracellular BMP-binding proteins like Sog and Cv-Tsg2 can increase short-range signaling near the Dpp-expressing LVs, but decrease long-range signaling from the LVs into the PCV region [10].

As a more rigorous test of cell autonomy, we generated large homozygous *gyc76C* ^*3L043*^ or *gyc76C* ^*KG0373ex33*^ clones using the *Minute* technique and *hs-FLPase*, examining these at 28 hours AP which, because of the slowed development of *M* ^-^/+ flies, corresponds to approximately 24 hours AP in wild type flies. Clones that encompassed the region of PCV formation on both the dorsal and ventral epithelia could result in the complete or near-complete loss of pMad from the PCV (S8A, S8A’, S8C and S8C’ Fig. Effects of additional *gyc76C* and *for* mutant clones on PCV development). However, individual PCV cells within smaller clones often had pMad levels identical to those in neighboring heterozygotic cells (Fig 3J and 3K’, S8B and S8B’ Fig). We observed similar non-autonomy in *for* ^*02*^ mutant clones in 24 hour AP or older wings (Fig 3L and 3L’); *for* ^*02*^ clones could even occasionally disrupt PCV formation in neighboring *for*/+ or +/+ cells (S8D and S8E Fig). These non-autonomous effects are quite similar to those caused by clones lacking the extracellular BMP-binding regulators Sog, Cv-Tsg2 and Cv-2 [10, 15].

The overexpression of Gyc76C also induced ectopic venation and anti-pMad staining in a non-autonomous fashion. *hh-Gal4* expression is limited to the posterior of the wing (Fig 2K), but *hh-Gal4*-driven expression of *UAS-myc-gyc76C* resulted in ectopic venation in the anterior compartment of adult wings (Fig 2J), and ectopic pMad anterior to the region of Gyc76C overexpression in pupal wings (Fig 3M and 3N).

### Gyc76 interacts genetically with Sog and other extracellular regulators of BMP signaling

The clonal analyses above strongly suggest that Gyc76C and For do not regulate BMP signaling in the pupal wing at the level of cell-autonomous signal transduction, but rather influence the extracellular regulation of BMP secretion, movement or reception. We therefore next examined the roles of BMPs and BMP-binding proteins in Gyc76C activity using genetic interactions. In the results that follow, at least 10 wings of each genotype were compared, and results were identical in all of them.

First, Gyc76C can act downstream of Dpp expression. Overexpression of *UAS-dpp-GFP* using an L5-specific Gal4 driver [75] expanded the width of the adult L5 (S7H Fig), but co-expression of *UAS-Gyc76C-RNAi* in L5 significantly reduced this expansion (S7I and S7J Fig).

**Fig 4:**
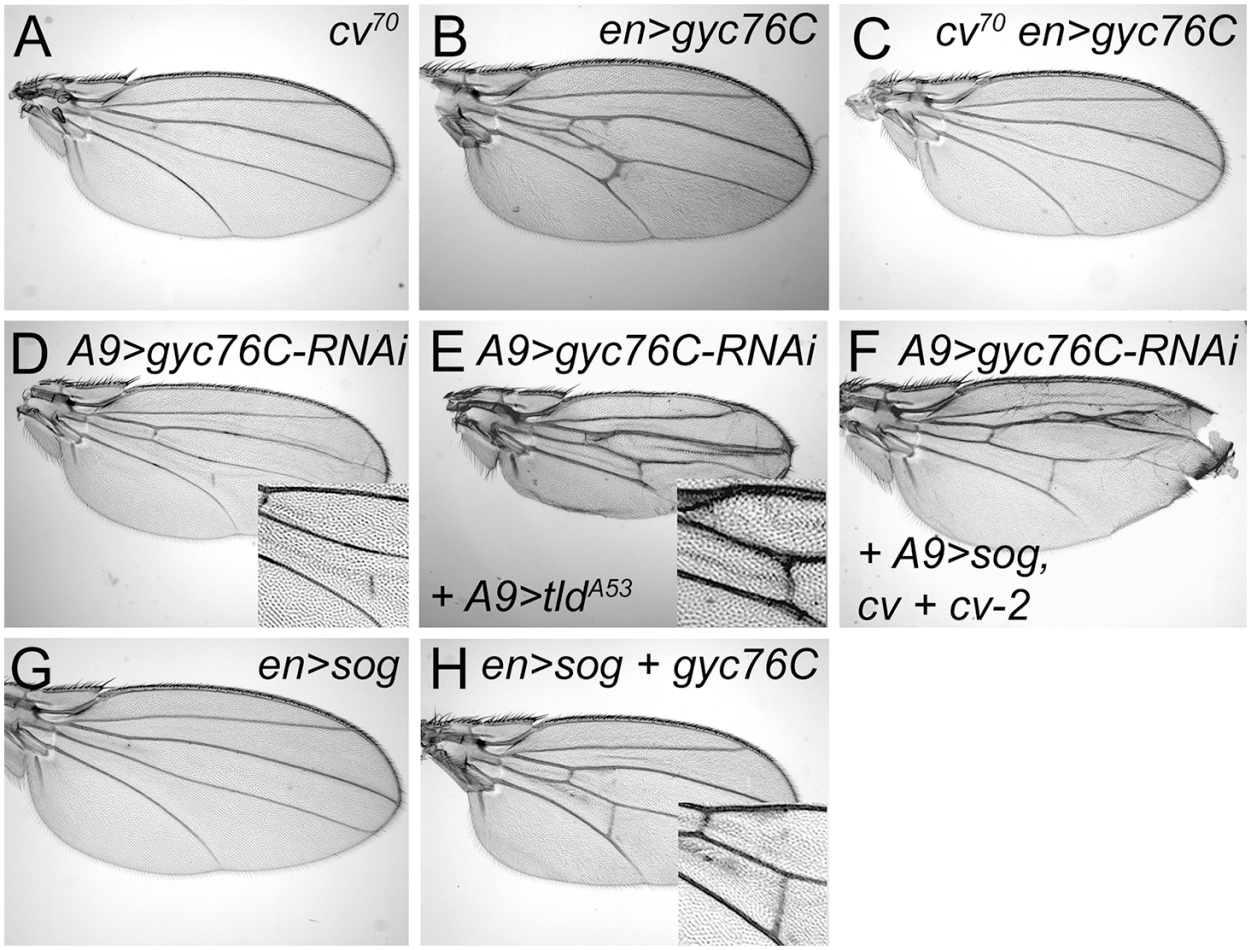
Genetic interactions between *gyc76C* and extracellular modifiers of BMP signaling. (A) Complete loss of the PCV in *cv* ^*70*^ wing. (B) Ectopic venation in *en-Gal4 UAS-myc-gyc76C* / + wing. (C) Loss of PCV and lack of ectopic venation in *cv* ^*70*^ / Y; *en-Gal4 UAS-myc-gyc76C* / + wing. (D) PCV disruption in *A9-Gal4*/*+; UAS-gyc76C-RNAi* (VDRC v6552) / + wing. (E,F) Rescue of the PCV disruption normally caused by *gyc76C* knockdown by expression of an activated form of Tld in an *A9-Gal4* / *+; UAS-tld* ^*A53*^ / +; *UAS-gyc76C-RNAi* / + wing (E), or by overexpression *sog*, *cv* and *cv-2* in *A9-Gal4* / *cv* ^*EP1349*^; *cv-2* ^*EP1103*^ / +; *UAS-sog / UAS-gyc76C-RNAi* wing (F). (G) PCV loss in *en-Gal4 / +; UAS-sog / +* wing. (H) Rescue of PCV loss by Gyc76C overexpression in *en-Gal4 UAS-myc-gyc76C* / +; *UAS-sog* wing.

Gyc76C’s vein-promoting activity also depended on the presence of the secreted BMP binding protein Cv-Tsg2. Loss of Cv-Tsg2 prevents BMP signaling in the pupal PCV and thus PCV formation in adults [9–11] (Fig 4A). The ectopic venation normally caused by *en-Gal4 UAS-gyc76C* was blocked in a *cv* hemizygous background, and the overexpression of *gyc76C* did not rescue crossvein formation (Fig 4B and 4C).

However, altering the levels of any single BMP, BMP regulator or effector did not rescue the defects caused by moderate *gyc76C* knockdown. The PCV disruption caused by *A9-Gal4*-driven expression of *UAS-gyc76C-RNAi* (Fig 4D) was not improved by individual co-expression of *UAS-gbb-Flag*, *UAS-tlr*, *UAS-cv-His*, *cv* ^*EP1349*^, *UAS-myc-cv-2-V5*, *cv-2* ^*EP1103*^, *UAS-sog*, *UAS-tkv-HA*, *UAS-punt*, *UAS-mad-Flag* or *UAS-medea* (the sole *D*. *melanogaster* co-Smad).

Nonetheless, we found that greatly increasing Sog cleavage could counteract the PCV-disrupting effects. *en-Gal4*-driven overexpression of the Tlr protease rescued the crossveinless disruption of *ade5* ^*X1*^ mutants (S6G Fig). And while Tlr overexpression did not rescue PCV disruption in *A9-Gal4*, *UAS-gyc76C* ^*RNAi*^ wings, expressing an activated form of Tld (Tld^A53^) did (Fig 4E). This suggests that *gyc76C* knockdown decreases BMP signaling by increasing the affinity of the Sog/Cv-Tsg2 complex for BMPs.

The effects of *gyc76C* overexpression are consistent with this hypothesis. Strong overexpression of *UAS-sog* with *en-Gal4* always blocks PCV formation (Fig 4G); the BMPs can likely still move as part of the Sog/Cv-Tsg2 complex, but the excess Sog overwhelms the available Tlr and Cv-2 so that BMPs remain sequestered in the complex [8, 74]. Increasing Sog cleavage with Tlr overexpression can rescue the crossvein defects caused by Sog overexpression [13], as can overexpression of Cv-2 [8]. *en-Gal4*-driven expression of *UAS-gyc76C* also rescued the PCV loss normally caused by *UAS-sog* expression (Fig 4H).

Since Tsgs and Cv-2 can also decrease the BMP-sequestering activity of the Sog/Cv complex, we tested whether overexpression of Cv-Tsg2 or Cv-2 could rescue *gyc76C* knockdown in combination with each other or with Sog. The PCV disruption caused by *A9-Gal4*-driven expression of *UAS-gyc76C-RNAi* (Fig 4D) was not improved by co-expression of *cv* ^*EP1349*^, *cv-2* ^*EP1103*^ or *UAS-sog* in any single or pair-wise combination. It was rescued, however, by triple co-expression of *UAS-sog*, *cv* ^*EP1349*^, and *cv-2* ^*EP1103*^ (Fig 4F). This result cannot be explained if *gyc76C* knockdown simply increased the affinity of the Sog/Cv-Tsg2 complex for BMPs, since adding excess Sog should increase BMP sequestration, not reduce it. Rather, we hypothesize that *gyc76C* knockdown also reduces the movement of the Sog/Cv-Tsg2 complex into the PCV region. Excess Sog can overcome this defect in diffusion, but only increases BMP signaling in a genetic background (excess Cv-Tsg2 and Cv-2) that frees BMPs from the excess Sog. In summary, our results suggest that Gyc76C knockdown has complex effects on Sog function, both increasing Sog’s affinity for BMPs, but also decreasing the range of Sog movement (see Discussion).

### Gyc76C and For affect the wing extracellular matrix

The non-autonomous, complex effects of Gyc76C and For on BMP signaling and Sog function are reminiscent of similar effects caused by altering the ECM in different developmental contexts (see Discussion). Moreover, the adult wing blistering caused by very strong loss of Gyc76G activity (Fig 2H detail) suggests a failure to properly adhere the two wing epithelia, an effect that can also be caused by altering the wing ECM and its receptors. Gal4-driver overexpression mediated by a UAS-containing *EP* insertion near the *for* locus was also reported to induce blistering [76]. We therefore examined the effects of cGMP activity on the levels and distribution of the ECM components Collagen IV using the 6G7 monoclonal antibody, LamininB2 (LanB2, also called Lanγ1) using anti-LanB2, and the secreted perlecan heparan sulfate proteoglycan (HSPG) Terribly reduced optic lobes (Trol, previously named *l(1)zw1*) using anti-Trol and a *trol-GFP* protein trap. In the normal pupal wing all three of these formed a diffuse ECM with scattered laminar aggregates; the aggregates were especially prominent with the 6G7 anti-Collagen IV. ECM proteins also concentrate in the hemocytes that circulate between the wing epithelia, and anti-LanB2 also stained the apical surfaces of the epithelia.

**Fig 5:**
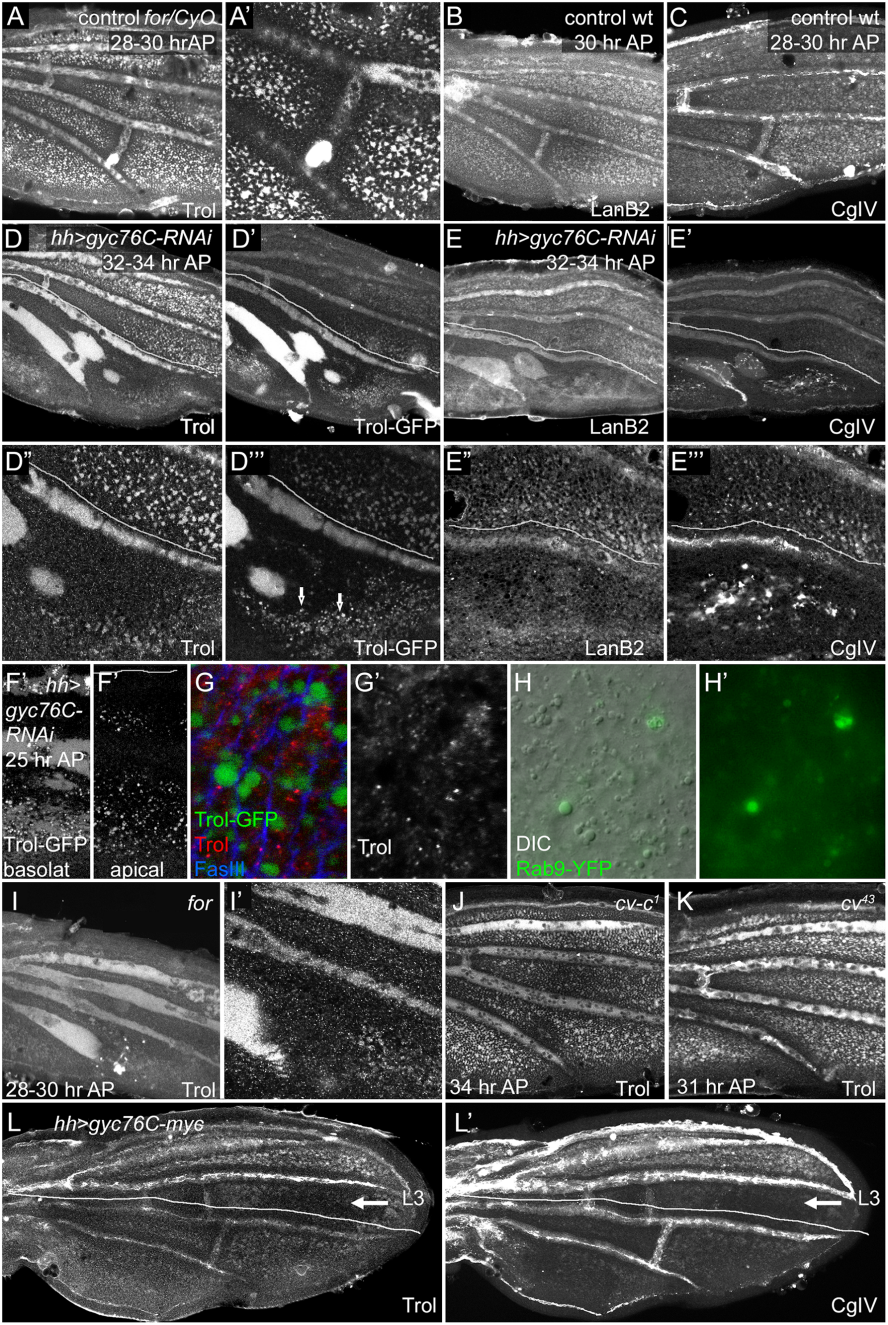
Effects of *for* and *gyc76C* on wing ECM. Lines in (D-E”‘, L-L’) show approximate limits of posterior *hh-Gal4* expression. (A,A’) Anti-Trol (Perlecan) staining in control *for* ^*02*^/*CyO*, *Tb* wing at 28–30 hours AP. (B-C) Anti-LanB2 (B) and 6G7 anti-Collagen IV (CgIV) staining in control wild type wings at 28 hours AP (B) and 28–30 hours AP (C). Controls show normal ECM concentration in veins and intervein pockets in both anterior and posterior. (D-E”‘) Anti-Trol staining (D,D”), Trol-GFP (D’,D”‘), Anti-LanB2 (E,E”) and 6G7 anti-Collagen IV (CgIV) (E’,E”‘) staining in 32–34 hour AP *hh-Gal4 UAS-gyc76C-RNAi* wings. Posterior ECM is more diffuse in LVs and depleted from intervein pockets. In addition, Trol-GFP accumulates more strongly in L5 and PCV region, and is also found in vesicular puncta (arrows in D”‘) in interveins, and CgIV accumulated in abnormal aggregates in veins and interveins (E’,E”‘). (F-G’) 24 hours AP *trol-GFP; hh-Gal4 UAS-gyc76C-RNAi* wings. (F,F’) Posterior GFP puncta are visible in both basal and apical focal planes. (G,G’) High magnification images of wings stained with anti-Trol (red, white) and anti-FasIII (blue) to visualize cell outlines. GFP puncta are intracellular and do not stain with anti-Trol. (H,H’) Detail of posterior intervein region of *hh-Gal4 UAS-gyc76C-RNAi UAS-rab9*.*YFP* wing at 24 hours AP, showing abnormally large vesicles (H, DIC optics) that co-localize with Rab9.YPF fluorescence (H,H’). (I,I’) Anti-Trol staining in 28–30 hour AP *for* ^*02*^ homozygote, aged and stained at same time as control in (A). Trol is broader and more diffuse in LVs, lost from PCV region, and depleted from intervein pockets. (J,K) Normal-appearing anti-Trol staining in *cv-c* ^*1*^ (J) and *cv* ^*43*^ (K) wings, except for loss of PCV. (L,L’) anti-Trol (L) or 6G7 anti-CgIV (L’) staining 28–30 hour AP *hh-Gal4 UAS-gyc76C-myc* wing. Intervein staining in the posterior is more diffuse and depleted from intervein pockets; this defect extends anterior to the region of *hh-Gal4* expression (lines) into intervein between L3 and L4 (arrow). Staining is normal anterior to L3.

We did not detect gross histological changes in the ECM after *gyc76C* knockdown prior to 24 hours AP, although the more open, pocket-like architecture of younger pupal wings makes it more difficult to detect ECM organization at this stage. Profound defects appeared, however, around 24–28 hours AP and strengthened from 28–34 hours AP, appearing slightly earlier in *for* mutants (Fig 5 and S9G–S9J’ Fig). By 24 hours AP the ECM normally fills both the large vein channels and smaller basolateral pockets between cells in intervein regions (Figs 1B and 5A–5C). Since the ECM is prominent in the normal veins, the PCV loss and LV expansion in *for* mutant wings, or in the posterior *hh-Gal4 UAS-gyc76C-RNAi* wings, caused parallel losses or gains of vein ECM (Fig 5D–5E”‘, 5I and 5I’). However, we also observed histological changes in the organization of the ECM that were not simply reflections of altered venation.

First, the basolateral pockets of intervein ECM, although initially normal, were progressively depleted in the posterior after posterior *gyc76C* knockdown (Fig 5D–5E”‘), or throughout the wing in *for* homozygotes (Fig 5I and 5I’) (*for* time course in S9G–S9J’ Fig). As this occurred the vein ECM became broader and more diffuse, and was often retained in abnormal vein-like blobs near the site of the PCV. The broadened ECM accumulation near veins did not strictly correlate with altered vein specification: the extremely broad L5 ECM caused by posterior *gyc76C* knockdown extended into regions lacking vein markers such as heightened pMad or reduced DSRF, and the vein-like blobs near the normal PCV site were retained after molecular markers of PCV development vanished (S9A and S9A’ Fig). In *hh-Gal4 UAS-gyc76C-RNAi* wings diffuse Trol-GFP was especially strong in L5 and the PCV-like blobs (Fig 5D”‘). 6G7 anti-Collagen IV staining showed an abnormally high accumulation of laminar aggregates in the posterior of *hh-Gal4 UAS-gyc76C-RNAi* wings (Fig 5E”‘ and S9J Fig).

These gross organizational defects were preceded by a more subtle change: at 24 hours AP abnormally large intracellular vesicles appeared in the interveins (Fig 5D”‘ and 5F–5H’, S9B–S9F Fig). These containing Trol-GFP and were likely endocytotic, since many co-localized with the late endocytic marker Rab9.YFP (Fig 5H and 5H’), although Trol-GFP only rarely overlapped the late endocytic marker Rab7, and did not significantly overlap the early endocytic marker Rab5, or the recycling vesicle marker Rab11 (S9C–S9E’ Fig). Intriguingly, anti-Trol staining did not accumulate in the GFP-containing vesicles (Fig 5G and 5G’) or co-localize with Rab9.YFP (S9B and S9B’ Fig). Since the GFP tag in Trol-GFP is inserted into the N terminal domain II, while the anti-Trol was produced against the C-terminal domain V [77], it is possible that the GFP represents uptake of an abnormal cleavage product of Trol-GFP. We will present results below suggesting that the vesicles are a cellular reaction to breakdown of the ECM.

The ECM phenotypes are not a general result of changes in BMP signaling or a crossveinless condition. *cv* null mutations block most or all PCV BMP signaling during the initial stages of PCV formation and broaden signaling in the LVs [9–11], but did not obviously alter wing ECM outside the missing PCV (Fig 5K and S9L Fig). Nor did we detect ECM defects outside the missing PCV in crossveinless wings mutant for the Rho-Rac GAP Crossveinless c (*cv-c* ^*1*^) or expressing *en-Gal4 UAS-dys-RNAi* (Fig 5J, S9M and S9N Fig).

The effects of cGMP activity on the ECM were not strictly cell autonomous. Moderate-sized *gyc76C* ^*3L043*^ or *for* homozygous clones did not deplete the intervein pockets or increase accumulation of Collagen IV aggregates, even where dorsal and ventral clones overlapped (S9K and S9K’ Fig). Posterior overexpression of *gyc76C* with *hh-Gal4* also altered wing ECM in a non-autonomous fashion. While ECM in the LVs appeared normal, ECM in the intervein pockets was fainter and more diffuse in the posterior; this effect extended up to L3, well anterior of the region of *hh-Gal4* expression (Fig 5L and 5L’).

### Gyc76C regulates matrix metalloproteinase levels

Since reductions in Gyc76C or For activity disrupt several components of the wing ECM, we next searched for effects on components known to organize or modify the ECM. Posterior *gyc76C* knockdown did not cause posterior-wide changes in the levels of ECM receptors such as the glypican Dlp, Dystroglycan, or the integrins Mys, Mew and If, nor alter expression of the *mys* expression regulator Delilah [78]. Nor could we detect posterior-wide changes in the BMP receptor Thickveins or the vein-width regulator Notch. Changes were limited to those caused by altered venation, and only for those proteins whose levels are normally different in vein and intervein (S10 Fig).

**Fig 6:**
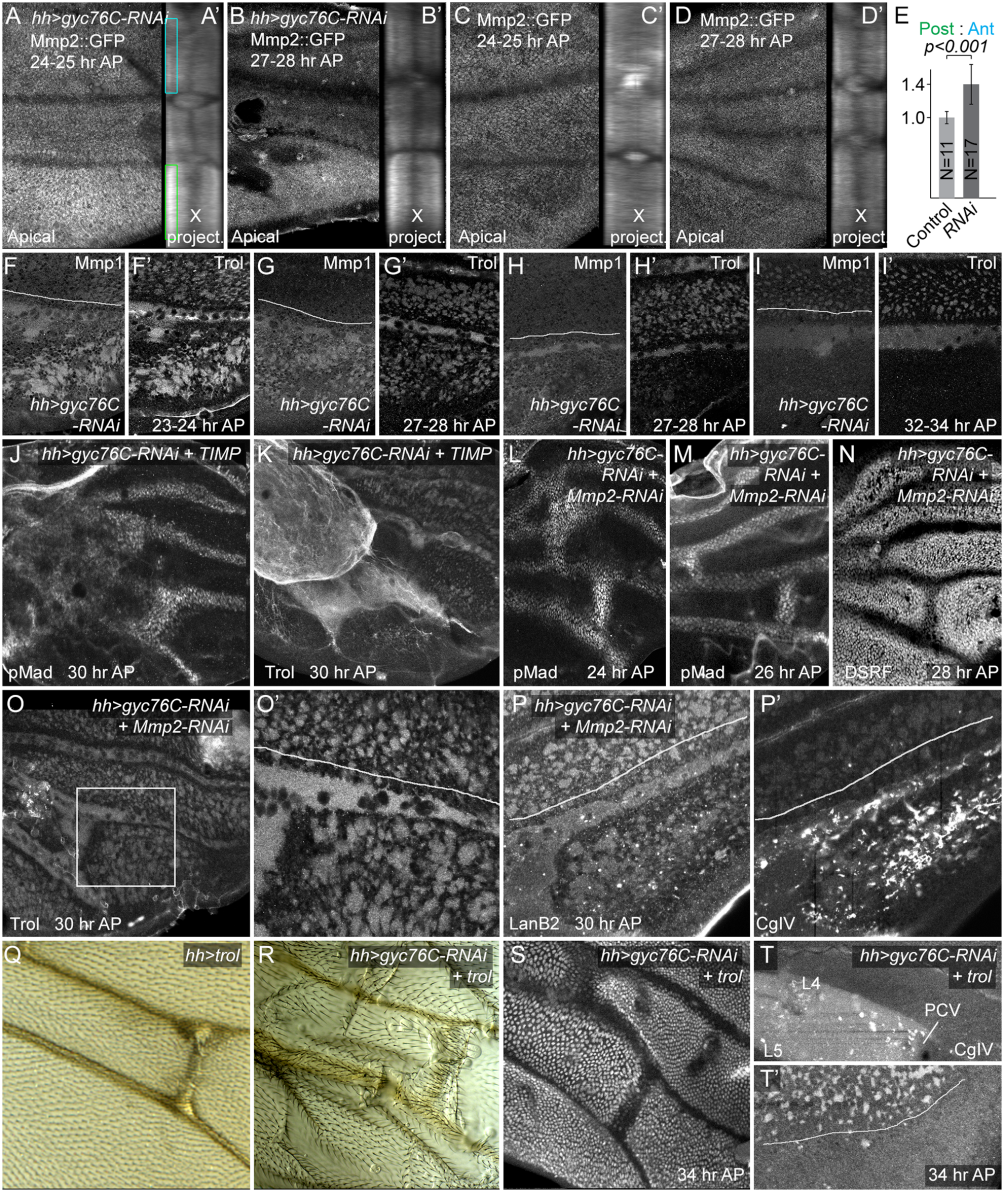
Matrix metalloproteinase and ECM involvement in *hh-Gal4 UAS-gyc76C-RNAi* phenotypes. (A-D’) Increased posterior MMP2::GFP levels (anti-GFP staining) in *gyc76C* knockdown wings (A-B’), compared with wild type (C-D’), at 24–25 hours AP (A,A’,C,C’) and 27–28 hours AP (B,B’,D,D’). The posterior increase was most obvious in apical focal planes (apical) and in projections of cross-sections summed along the proximo-distal axis of the image (X project.). (E) Ratios of posterior to anterior Mmp2::GFP intensity in apical portions of epithelia, corresponding to boxes in (A), in 23–25 hour AP Mmp2::GFP (Control) and *hh-Gal4 UAS-gyc76C-RNAi* (RNAi) wings. Error bars show standard deviation. The ratio was significantly higher in RNAi wings at p<0.001, using single-tailed Student’s T and Mann-Whitney tests. (F-I’) Increased anti-MMP1 levels in posterior veins and interveins of *gyc76C* knockdown wings at 23–28 hours AP, even as Trol levels decrease in posterior intervein pockets at 27–28 hours AP (F-H’). At 32–24 hours AP, when Trol is completely lost from intervein pockets, so is Mmp1 (I,I’). See S9S–S9X Fig for lower magnification images and anti-Mmp1 in control wings. (J,K) Rescue of PCV in *gyc76C* knockdown wings by expression of *UAS-TIMP*, as detected with anti-pMad (H) or anti-Trol (I). Ectopic pMad in proximal wing correlates roughly with the proximal region where abnormal levels of Trol accumulate. (L-P’) Rescue of PCV and ECM abnormalities in *gyc76C* knockdown wings by expression of *UAS-Mmp2-RNAi*. (L-N) Nearly normal PCVs, detected using anti-pMad (L,M) and anti-DSRF (N) at 24, 26 and 28 hours AP, respectively. (O-P’) Nearly normal anti-Trol (O,O’) and anti-LanB2 (P) staining in posterior veins and intervein pockets at 30 hours AP, although posterior has abnormal aggregates of 6G7 anti-CgIV staining (P’). (Q-T’) Effects of overexpression of *UAS-trol* (EP insertion into *trol* locus). (Q) Broadening of adult PCV caused by *hh-Gal4- UAS-trol*. (R) Rescue of adult PCV in *gyc76C* knockdown wing by *UAS-trol*, despite increased wing blistering. (S) Rescue of PCV in 34 hour AP *gyc76C* knockdown wing by *UAS-trol*, as assayed by downregulation of DSRF. (T,T’) Abnormal accumulation of diffuse 6G7 anti-CgIV staining proximal to PCV between L4 and L5 (T), and failure to rescue posterior intervein pockets (2.4x magnification detail, T’) after *UAS-trol* expression in 34 hour AP *gyc76C* knockdown wing.

The depletion of ECM from the interveins and the diffuse ECM appearance the veins, next suggested the involvement of the extracellular matrix metalloproteinases (Mmps). This is also consistent with the possible appearance of a Trol-GFP cleavage product noted above, as vertebrate perlecan can be cleaved by Mmps [79]. There are two *D*. *melanogaster* Mmps: Mmp2, which is predicted to be GPI-linked to the cell surface, and Mmp1, which is diffusible [80–82]. An engineered Mmp2::GFP produced by the endogenous *Mmp2* locus [83] is normally expressed in a slightly patchy pattern in intracellular structures and the cell cortex, more weakly in vein cells and more strongly near the wing hinge; the cytoplasmic GFP is stronger apically (Fig 6C and 6D’), without any strong anterior-posterior bias (11 of 11 23–25 hour AP wings). After posterior *hh-Gal4*-driven knockdown of Gyc76C, posterior Mmp-2 levels were higher and more uniform, an effect especially noticeable in more apical focal planes, and after a summing projection of all cross-sections along the proximo-distal (X) (Fig 6A and 6B’). Posterior apical Mmp2::GFP was increased by 25% or greater over anterior in 13 out of 16 23–25 hour AP wings, and the increase was significant in a comparison of all experimental and control wings (Fig 6E). Those wings lacking the effect may reflect variation in its timing.

Anti-Mmp1 staining in normal pupal wings was largely extracellular and concentrated in the diffuse ECM of veins and intervein pockets. *hh-Gal4 UAS-gyc76C* always increased the posterior levels of Mmp1 in the ECM of both veins, especially L5 and PCV-like blobs, and intervein pockets beginning at 24 hours AP (details in Fig 6F–6I’; low magnification in S9S–S9U Fig; anti-Mmp1 in control wings in S9W and S9X Fig) This increased staining was retained in the veins but was transient in the intervein pockets: as the intervein pockets became depleted of ECM beginning at 27–28 hours AP, Mmp1 levels in the pockets also decreased, although increased Mmp1 was sometime still observed in pockets partially depleted of anti-Trol (Fig 6H and 6H’). The increased Mmp1 is likely due to changes in Mmp1 secretion or accumulation rather than transcription, as an *Mmp1-lacZ* enhancer reporter that reproduces *Mmp1* expression in other contexts [84] was not obviously altered in *hh-Gal4 UAS-gyc76C-RNAi* wings (S9Q and S9R Fig). Given the strong association of Mmp1 with wing ECM, Mmp1’s abnormal accumulation in the abnormal ECM of *gyc76C* knockdown wings could be both a result and a cause.

### Reducing Mmp activity largely rescues the effects *gyc76C* knockdown on the ECM and BMP signaling

To test the role of Mmp activity in the ECM and BMP signaling changes caused by reduced Gyc76C activity, we first overexpressed the *D*. *melanogaster* member of the diffusible Tissue inhibitor of metalloproteases (Timp) protein family, which can inhibit both Mmp1 and Mmp2 activities [85, 86]. *hh-Gal4 UAS-gyc76C-RNAi UAS-Timp* pupae did not produce adults and pupal wings were foreshortened, likely due to Timp’s effects on wing disc eversion [87]. Nonetheless, Timp greatly improved PCV development in *hh-Gal4 UAS-gyc76C-RNAi* pupal wings, forming normal or nearly normal PCVs as assessed by heightened pMad or reduced DSRF pMad in 9/9 28 hour AP or older wings (Fig 6J). *hh-Gal4*-driven expression of *UAS-Timp* in a *gyc76C* knockdown background (Fig 6K) also increased the ECM, but largely in an in an abnormal proximal clump likely caused by excess inhibition of Mmp activity. Intriguingly, BMP signaling also expanded in the proximal wing adjacent to the excess ECM (Fig 6J).

We next tested the roles of the Mmps individually using RNAi lines with proven efficacy [84]. *UAS-Mmp1-RNAi* did not improve the defects of *hh-Gal4 UAS-gyc76C-RNAi* wings, but *UAS-Mmp2-RNAi* did. *hh-Gal4 UAS-gyc76C-RNAi UAS-Mmp2-RNAi* larvae and pupae were occasionally unhealthy-appearing, generating fragile wings with poor morphology and development, but the ECM in those that were healthy appeared almost normal: the width and strength of the staining around posterior veins and the strength and number of intervein ECM pockets were nearly normal (Fig 6O and 6P). The abnormally large intracellular vesicles normally found in the interveins of *gyc76C* knockdown wings were also greatly reduced (S9Y and S9Z Fig), suggesting that these are a cellular reaction to Mmp-induced breakdown of the ECM. While 6G7 anti-Collagen IV staining still showed the abnormally high numbers of Collagen IV aggregates observed in *hh-Gal4 UAS-gyc76C-RNAi* wings (Fig 6P’), it should be noted that *hh-Gal4 UAS-Mmp2-RNAi* in otherwise wild type wings also increases abnormal anti-Collagen IV aggregates in the posterior (S9O and S9P Fig). BMP signaling in the PCVs was also largely rescued, as assessed by heightened pMad or reduced DSRF in 15/17 24–32 hour AP wings (Fig 6L–6N). These results strongly suggest that most of the ECM and BMP signaling defects caused by reduced Gyc76C activity are caused by increased Mmp activity.

We next asked whether the ECM and BMP signaling defects were linked, or were independent effects of Mmp activity, by looking at the effects of manipulated the ECM directly. It is difficult to remove most ECM components from the wing: null mutants are lethal or cause morphological abnormalities at earlier stages, and mosaic techniques cannot be used for those ECM components, like Trol and Collagen IV, that are likely transported into the wing via hemolymph and hemocytes [88, 89]. Instead, we tested the ability of overexpressed ECM to rescue the effects of *gyc76C* knockdown, choosing Trol because *hh-Gal4*-driven expression of *UAS-trol* in a wild type background led to few abnormalities beyond a slight broadening of the PCV (Fig 6Q). *hh-Gal4*-driven expression of both *UAS-trol* and *UAS-gyc76C-RNAi* increased the blistering sometimes observed in adult wings after *gyc76C* knockdown, but partially or wholly rescued PCV formation in adult (Fig 6R) and pupal wings (6/7 28 hours AP or older; Fig 6S). Unlike *UAS-Mmp2-RNAi*, the very strong overexpression caused by *UAS-trol* did not obviously improve the other ECM components in the posterior of *hh-Gal4 UAS-gyc76C-RNAi* pupal wings, but did cause an abnormal accumulation of diffuse 6G7 anti-CgIV staining between L4 and L5 proximal to the PCV (Fig 6T and 6T’), leaving open the possibility that the rescue was mediated by reorganization of the ECM, rather than by Trol directly.

## Discussion

**Fig 7:**
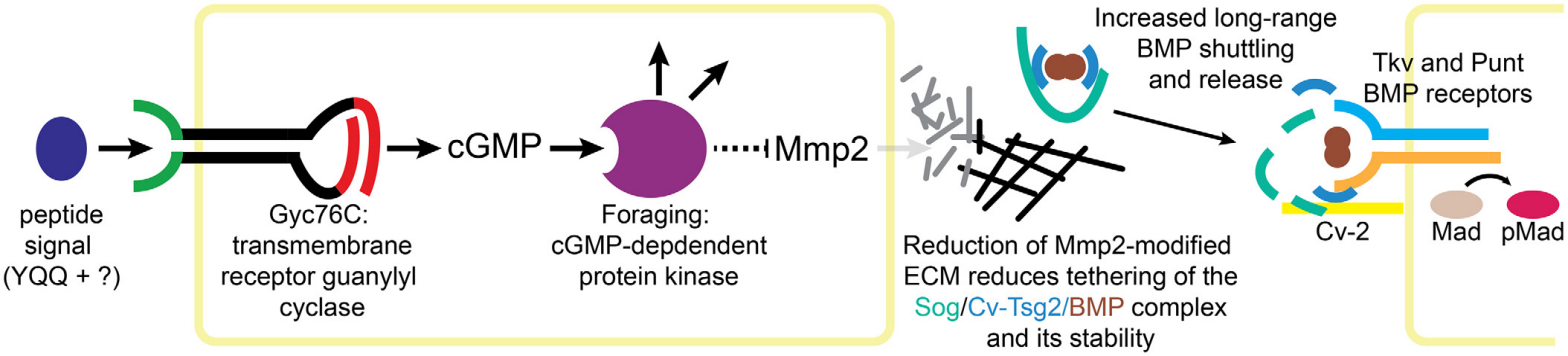
Model of Gyc76C and For in Mmp2-mediated ECM organization and BMP signaling. Peptide activation of Gyc76C stimulates cGMP production, activating For, leading to the repression of Mmp2 activity. Reduced Mmp2 activity decreases ECM reorganization, decreasing tethering of the Sog/Cv-Tsg2/BMP complex to the ECM and reducing the stability of the complex, increasing long-range movement of the complex and the release of BMPs for signaling, activating the BMP receptors Tkv and Punt and their phosphorylation of Mad.

Here we report that mutation *3L043*, uncovered by a genetic screen to identify homozygous lethal mutations required for PCV development, is a novel allele of *gyc76C*, a transmembrane peptide receptor that, like vertebrate NPRs, acts as a guanylyl cyclase. We further show that *gyc76C* is likely linked by cGMP production to the activity of the cGMP-dependent kinase For, and that Gyc76C and For define a new pathway for the regulation of wing ECM (Fig 7). This pathway appears to act largely through changes in the activity of ECM-remodeling Mmp enzymes. Loss of *gyc76C* or For alter both the organization of the wing ECM and the levels of the two *D*. *melanogaster* Mmps, and the *gyc76C* knockdown phenotype can be largely reversed by knockdown of Mmp2. This is the first indication of a role for cGMP, Gyc76C and For function in the developing wing, and their effects on the ECM provides a novel molecular output for each.

We have also shown that Gyc76C and For are necessary for the normal refinement and maintenance of long-range BMP signaling in the posterior crossvein region of the pupal wing; in fact, crossvein loss is the most prominent aspect of the adult *gyc76C* knockdown phenotype. Our evidence suggests that this effect is also mediated by changes in Mmp activity, and most likely the Mmp-dependent reorganization of the ECM (Fig 7). In fact, our analysis using genetic mosaics finds no evidence for a reliable, cell autonomous effect of cGMP activity on BMP signal transduction in the wing. Thus, this apparent crosstalk between receptor guanylyl cyclase activity and BMP signaling in the wing is mediated by extracellular effects.

### cGMP, Mmp and BMP/TGFβ in mammals

It is noteworthy that the cGMP activity mediated by NPR or nitric oxide signaling can change also Mmp gene expression, secretion or activation in many different mammalian cells and tissues (e.g. [42–47]). Both positive and negative effects have been noted, depending on the cells, the context, and the specific Mmp. Given the strong role of the ECM in cell-cell signaling, the contribution of cGMP-mediated changes in Mmp activity to extracellular signaling may be significant.

There is also precedent for cGMP activity specifically affecting BMP and TGFβ signaling in mammals. cGMP-dependent kinase activity increases BMP signaling in C2C12 cells, and this effect has been suggested to underlie some of the effects of nitric oxide-induced cGMP on BMP-dependent pulmonary arterial hypertension [50, 52]. Conversely, atrial natriuretic peptide stimulates the guanylyl cyclase activities of NPR1 and NPR2 and can inhibit TGFβ activity in myofibroblasts; this inhibition has been suggested to underlie the opposing roles of atrial natriuretic peptide and TGFβ during hypoxia-induced remodeling of the pulmonary vasculature [48, 49, 51]. However, unlike the pathway we observed in the fly wing, these mammalian effects are thought to be mediated by the intracellular modulation of signal transduction, with cGMP-dependent kinases altering BMP receptor activity or the phosphorylation and nuclear accumulation of receptor-activated Smads [50, 51]. Nonetheless, it remains possible that there are additional layers of regulation mediated through extracellular effects, underscoring the importance of testing cell autonomy.

### Gyc76C and For in other contexts

Aside from its role in adult Malpighian tubule physiology, Gyc76C was previously shown to have three developmental effects: in the embryo it regulates the repulsive axon guidance mediated by Semaphorin 1A and Plexin A [34, 39], the proper formation and arrangement of somatic muscles [37], and lumen formation in the salivary gland [38]. All these may have links to the ECM. Loss of *gyc76C* from embryonic muscles affects the distribution and vesicular accumulation of the βintegrin Mys [37], and reduces laminins and the integrin regulator Talin in the salivary gland [38]. The axon defects likely involve a physical interaction between Gyc76C and semaphorin receptors that affects cGMP levels [39]; nonetheless, *gyc76C* mutant axon defects are very similar to those caused by loss of the perlecan Trol [90].

The parallels between the different contexts of Gyc76C action are not exact, however. First, only the wing phenotype has been linked to a change in Mmp activity. Second, unlike the muscle phenotype, the wing phenotype is not accompanied by any obvious changes in integrin levels or distribution, beyond those caused by altered venation (S10 Fig). Finally, most *gyc76C* mutant phenotypes are reproduced by loss of the Pkg21D (Dg1) cytoplasmic cGMP-dependent kinase [35, 37, 38, 91], instead of For (Dg2, Pkg24A) as found in the wing, and thus may be mediated by different kinase targets.

For has been largely analyzed for behavioral mutant phenotypes [92], and the overlap between Pkg21D and For targets is unknown. While many targets have been identified for the two mammalian cGMP-dependent kinases, PRKG1 (which exists in alpha and beta isoforms) and PRKG2, it is not clear if either of these is functionally equivalent to For. One of the protein isoforms generated by the *for* locus has a putative protein interaction/dimerization motif with slight similarity to the N-terminal binding/dimerization domains of alpha and beta PRKG1, but all three For isoforms have long N-terminal regions that are lacking from PRKG1 and PRKG2. In fact, a recent study suggested that For is instead functionally equivalent to PRKG2: Like PRKG2, For can stimulate phosphorylation of FOXO, and is localized to cell membranes in vitro [93]. But For apparently lacks the canonical myristoylation site that is thought to account for the membrane localization and thus much of the target specificity of PRKG2. FOXO remains the only identified For target, and *foxo* null mutants are viable with normal wings [94].

### The regulation of long-range BMP signaling by candidate Mmp2 targets

The loss of long range BMP signaling in the PCV region caused by knockdown of *gyc76C* can, like the ECM, be largely rescued by knockdown of Mmp2. Two results suggest that it is the alteration to the ECM that affects long-range BMP signaling, rather than some independent effect of Mmp2. First, the BMP signaling defects caused by *gyc76C* knockdown were rescued by directly manipulating the ECM through the overexpression of the perlecan Trol. Second, when Mmp activity is inhibited by overexpression of the diffusible Mmp inhibitor TIMP, this not only rescued the PCV BMP signaling defects caused by *gyc76C* knockdown, but also led to ectopic BMP signaling, not throughout the region of TIMP expression, but only in those regions with abnormal accumulation of ECM.

The Mmp2-mediated changes in the ECM likely affect long-range BMP signaling by altering the activity of extracellular BMP-binding proteins, particularly Sog. The BMPs Dpp and Gbb produced in the LVs bind Sog and Cv-Tsg2, shuttle into the PCV region, and are released there by Tlr-mediated cleavage of Sog and transfer to Cv-2 and the receptors (see Introduction and Fig 1B). Our genetic interaction experiments suggest that knockdown of *gyc76C* both increases Sog’s affinity for BMPs and reduces the movement of the Sog/Cv-Tsg2/BMP complex into the crossvein region.

Collagen IV provides the best-studied example for how the ECM might affect Sog activity. The two *D*. *melanogaster* collagen IV chains regulate BMP signaling in other contexts, and they bind both Sog and the BMP Dpp [25–27]. Results suggest that collagen IV helps assemble and release a Dpp/Sog/Tsg shuttling complex, and also recruits the Tld protease that cleaves Sog cleavage and releases Dpp for signaling [25, 27, 28]. *D*. *melanogaster* Mmp1 can cleave vertebrate Collagen IV [80]. Since reduced Gyc76C and For activity increases abnormal Collagen IV aggregates throughout the wing and diffuse Collagen IV in the veins, we hypothesize that these Collagen IV changes both foster the assembly or stability of Sog/Cv-Tsg2/BMP complexes and tether them to the ECM, favoring the sequestration of BMPs in the complex and reducing thelong-range movement of the complex into the region of the PCV (Fig 7).

While few other *D*. *melanogaster* Mmp targets have been identified, it is likely that Mmp1 and Mmp2 share the broad specificity of their mammalian counterparts [80, 81], so other ECM components, known or unknown, might be involved. For instance, vertebrate Perlecan and can be cleaved by Mmps [79]. Trol regulates BMP signaling in other *D*. *melanogaster* contexts [29, 30], and Trol overexpression rescue *gyc76C* knockdown’s effects on BMP signaling. But while null *trol* alleles are lethal before pupal stages, normal PCVs were formed in viable and even adult lethal alleles like *trol* ^*G0023*^, and *actin-Gal 4*-driven expression of *trol-RNAi* using any of four different *trol-RNAi* lines did not alter adult wing venation. Loss of the *D*. *melanogaster* laminin B chain shared by all laminin trimers strongly disrupts wing venation [24], and a zebrafish laminin mutation can reduce BMP signaling [95].

Finally, it was recently shown that Dlp, one of the two *D*. *melanogaster* glypicans, can be removed from the cell surface by Mmp2 [96]. While *gyc76C* knockdown did not detectably alter anti-Dlp staining in the pupal wing (S10G and S10H Fig), it is noteworthy that Dlp and the second glypican Dally are required non-autonomously for BMP signaling in the PCV and that they bind BMPs and other BMP-binding proteins [15, 17].

## Methods

### *D*. *melanogaster* stocks

The following were generated from Bloomington *Drosophila* Stock Center stocks, unless otherwise indicated.

*A9-Gal4 w*

*y w; ap-Gal4 UAS-GFP/CyO*

*y w; en-Gal4*

*y w; en-Gal4 UAS-FLP*

*UAS-GFP; hh-Gal4/TM6,Tb*

*hh-Gal4 UAS-GFP/TM6*,*Tb* (recombinant generated in lab)

*L5-Gal4* (*3*.*7KX-lacZ/UAS*) kindly provided by J. de Celis [75]. *L5-Gal4 UAS-dpp-GFP* recombinant generated in lab.

*y w; UAS-FLP*

*y w hs-FLP; ubi-mRFP.nls FRT^40A^ /CyO*

*y w; FRT^2A^*

*y w hs-Flp; hs-GFP RpS17* ^*4*^ [also known as *M(3)i* ^*55*^] *FRT* ^*2A*^ */TM3*, *Sb* (kindly provided by G. Struhl)

*y w; FRT^82B^*

*y w*, *FRT* ^*82B*^, *RpS3* ^*Plac92*^[also known as *M(3)w* or *M(3)95A*] *ubi-GFP/TM6B*, *Tb*

*FRT* ^*82B*^ *dys* ^*EP3397*^ */TM6*,*Tb* (FRT recombinant kindly provided by D. Olson)

*gyc76C* ^*KG03723ex33*^ */TM3*,*Sb*, *UAS-myc-gyc76C* and *UAS-myc-gyc76* ^*D945A*^, kindly provided by A. Kolodkin [34]. *gyc76C* ^*KG03723ex33*^ *FRT* ^*2A*^, *en-Gal4 UAS-myc-gyc76C* and *hh-Gal4 UAS-myc-gyc76C* recombinants were lab-generated. Gyc76C overexpression experiments used a second chromosome *UAS-myc-gyc76C*, except for those of Fig 3M and 3N which used a lab-generated *for* ^*02*^; *hh-Gal4 UAS-myc-gyc76C* /*CyO-TM6*,*Tb* stock.

*y cv* ^*1*^ *v ade5* ^*1*^ *fl/FM6*, kindly provided by D. Clark [68]. Crossveinless males were crossed to *Df(1)ED7165*/*FM7h*; the deficiency covers *ade5* but not *cv*.

*y w; Nplp1^EY11089^*

*for* ^*02*^ */CyO*,*Tb*, *for* ^*K04703*^/*CyO*,*Tb*

*for* ^*02*^ *FRT* ^*40A*^/*CyO*, recombinant generated in lab.

*UAS-PDE6-RNAi*, *UAS-PDE6* and *UAS-PDE6* ^*C1128S*^ kindly provided by Dr. S. Davies [70, 71].

*cv* ^*43*^ [11]

*UAS-dpp-GFP*, *UAS-tlr-HA*, *UAS-tld* ^*A53*^, *UAS-sog-HA*, *cv* ^*70*^, kindly provided by M. O’Connor [10, 13].

*cv-2* ^*P(EP)1103*^ (Szeged Stock Center) [4]

*cv* ^*P(EP)1349*^; *cv-2* ^*P(EP)1103*^ *UAS-sog-HA* stock generated in lab.

*cv-c^1^*

*cv^P(EP)1349^*

*y w; P(UASp-YFP*.*Rab9)* [97]

*CG4839^MB10509^*

*UAS-gyc76C-RNAi*, *UAS-dys-RNAi* and *UAS-Pkg24-RNAi* lines were from the VDRC. *UAS-gyc76C RNAi; hh-Gal4/CyO-TM6*,*Tb* and *en-Gal4 UAS-dys-RNAi* stocks generated in lab.

*trol-GFP* (*P{PTT-un1}trol* ^*G00022*^) [98] from Kyoto DGRC.

*Mmp2*::*GFP/CyO*, kindly provided by J. Sun [83]

*UAS-TIMP*, *UAS-Mmp1-RNAi*, *UAS-Mmp2-RNAi* and *Mmp1-lacZ* kindly provided by D. Bohmann [84].

*P(GSV2)trol* ^*GS7407*^ (*UAS-trol*) kindly provided by J. Pastor-Pareja [88].

Bloomington deficiency kits and molecularly defined deletions and P element *w* ^+^ insertions for mapping.

### Mutagenesis

50 or more males were transferred to empty vials, allowed to dehydrate for 30 minutes, and then transferred overnight to new bottles contained filter paper soaked in a solution of 24mM EMS, 10Mm Tris pH 7.5 and 1% sucrose. The males were then transferred to dry tubes containing damp filter paper for 30 minutes before being crossed to 50 females for two days.

### Mapping

Mapping was as described in the Results, except that 3L043 lethality was initially mapped using Bloomington deficiency kits DK3,3L and DK3,3R (Bloomington), while *ade5* ^*X1*^ was initially mapped using meiotic recombination relative to molecularly mapped *w* ^*+*^ P element insertions [99]. The 3L044 missense mutation *gyc76C* ^*L635H*^ was identified by Sanger sequencing (University of Wisconsin-Madison Biotechnology Center) using primers 5’ ATGGATTGTTTGCCACCAACAG Fwd, and 5’ TCAAACAATCGGAATGAAGCTG Rev.

### Immunohistochemistry

Wing disc and pupal wing dissection, fixation and staining were as described previously [4, 8]; identical methods were used for larval CNS staining. Images were captured on BioRad and Olympus FV1000 confocal microscopes. Projections, cross-sections and quantifications were generated from Z-series images using ImageJ.

Concentrations and sources of primary antibodies were: 1:2000 rabbit anti-phosphoSmad3 (Epitomics); 1:500–1000 mouse anti-DSRF (Cold Spring Harbor Laboratory Antibody Facility); 1:50 mouse anti-Engrailed 4D9, mouse anti-FasIII or mouse anti-Mmp1 (Developmental Studies Hybridoma Bank); 1:500 rabbit anti-MTYamide [100] kindly provided by L. Schoofs; 1:500 rabbit anti-Trol [77] kindly provided by S. Baumgartner; 1:1000 rabbit anti-Drosophila Lamininγ1 (LanB2) (ABCAM); 1:50 mouse 6G7 anti-Collagen IV [22] kindly provided by J. Palka; 1:500 rabbit anti-GFP (MBL); 1:500 rabbit anti-Rab5 (ABCAM); 1:3000 rabbit anti-Rab7 [101] kindly provided by A. Nakamura; 1:1000 rabbit anti-Rab11 [102] kindly provided by D. Ready; 1:50 rabbit anti-Dei [78] kindly provided by A. Salzberg. Secondary staining used Jackson ImmunoResearch fluorescently-tagged (FITC, RITC, Cy2, Cy3, or Cy5) Min X anti-mouse IgG(H+L) or anti-rabbit IgG(H+L) antisera.

## Supporting Information

S1 Fig Additional venation mutations. Adult phenotypes caused by large homozygous posterior clones generated using *en-Gal4 UAS-Flp* and the *Minute* method.
(PDF)

S2 Fig Tandem duplication of *gyc76C* genomic region in BDGP sequence. Predicted duplicate gene names have the same color. The duplication has only been observed in a subset of the *iso-1* strain used for BDGP sequencing, and was likely caused by the mobilization of genomic DNA around an original *Doc* insertion [57]. When present, the aberration duplicates *CG14101* (termed *CG42529* in one duplicate) and the coding exons, but not promoter or 5’ UTR exons, of *gyc76C* (termed *GC42637* in one duplicate, although it is not certain that the longer *GC42637* primary transcript is made). The duplication is very unlikely to be present in the *3L043* chromosome: the duplication is not even present in all *iso-1* flies, and Canton-S and Oregon-R wild type strains lack both the *Doc* insertions and the duplication [57]. Since the *gyc76C* ^*L635H*^ mutation likely causes the *3L043* genotype, it is doubtful that the *3L043* chromosome contains a second, functioning duplication of *gyc76C* coding exons.
(PDF)

S3 Fig *ade5* mutants disrupt PCV development. (A) Mapping the *X1* mutation to the *ade5* region. *X1* does not lie at the other end of the deficiencies, since the proximal end of the BSC545 deficiency extends further than the proximal end of ED7165. (B) PCV disruption in *ade* ^*X1*^ wing. (C) PCV loss in *ade5* ^*1*^ wing. (D) PCV disruption in *ade5* ^*X1*^/*ade5* ^*1*^ wing. (E,F) anti-pMad staining is present in the PCV region of *ade5* ^*1*^ wings at 21 hours AP (E) but is largely lost by 25 hours AP (F). (G) *en-Gal4*-driven expression of *UAS-tlr* rescues the PCV in an *ade5* ^*X1*^ */Y* wing.
(PDF)

S4 Fig Nplp1 peptides are not required for full Gyc76C activity. (A) *Nplp1* locus. The P element insertion *Nplp1* ^*EY11089*^ lies in the second coding exon of Nplp1, placing stop codons between the region coding the N-terminal secretion signal peptide and the region coding neuropeptide precursors. (B,C) Larval CNS stained with antiserum against the Nplp1 peptide MTYamide. The strong staining in dorsal and ventral segmentally repeated neuronal cell bodies and axons in wild type (B) is missing in an *Nplp1* ^*EY11089*^ homozygote (C). (D,E) Two adult wings homozygous for *Nplp1* ^*EY11089*^. Unlike after *gyc76C* knockdown, the PCVs are present and complete, and occasionally show a small ectopic branch (D).
(PDF)

S5 Fig More Gyc76C knockdown effects on pupal venation. (A) Effects of dorsal *gyc76C* knockdown in 20 hour AP *ap-Gal4 UAS-GFP UAS-gyc76C-RNAi* wing. Top panels show that *ap*-driven GFP expression is limited to the dorsal epithelium. Middle panels show that anti-pMad staining is broader in the dorsal epithelium, both around the PCV and the LVs. Lower left panel shows overlay of dorsal (green) and ventral (red) pMad, with overlap in yellow. (B) In situ hybridization to 24 hour AP wing with *gyc76C* antisense probe. (C) Retention of anti-pMad in PCV of *hh-Gal4 UAS-gyc76C-RNAi* wing at 20 hours AP. (D,D’) 24 hour AP *en-Gal4 UAS-gyc76C-RNAi* wings showing loss of pMad (D) but suppression of DSRF (D’) in PCV.
(PDF)

S6 Fig Early disruption of PCV in *for* mutant. Comparison of wild type (A-A”‘) and *for* ^*02*^ homozygous (B-B”‘) pupal wings at 20 hours AP, with anti-pMad staining in red and anti-FasIII-stained cell membranes in green. (A,B) Low magnification image of single apical (nuclear) focal plane showing normal (A) and partially disrupted (B, arrow) pMad in PCV. (A’,B’) Low magnification image of basal focal plane, showing green membranes where dorsal and ventral epithelia have attached, and the dark basal lumen where epithelia have not yet attached. The lumen does not yet define physical veins in the region of the PCV. (A”,B”) Cross-sections (x) reconstructed from high magnification z-series images, along x lines in A’ or B’. Widths of normal (A”) or abnormal (B”) PCV pMad (red) regions are much narrower than the basal lumens. (A”‘,B”‘) High magnification projections of z-series images from all the pMad-containing focal planes on one epithelium, again showing PCV disruption (arrow) in the *for* mutant.
(PDF)

S7 Fig Effects of Gyc76C on adult vein width. (A,B) *L5-Gal4* (control) adult wing. Arrows demarcate the region of L5 posterior to the ACV, used in measurements of vein width. (C) L5 width in *hh-Gal4 UAS-GFP/+* flies is similar to wild-type and *L5-Gal4*. (D) Increased width of L5 in *hh-Gal4 UAS-GFP/ UAS-gyc76C-RNAi* (VDRC 6552) flies. (E) Comparison of L5 widths between wild type (wt), *hh-Gal4 UAS-GFP/+* and *hh-Gal4 UAS-GFP/UAS-gyc76C-RNAi* wings. (F and G) Normal L5 width in *L5-Gal4 UAS-gyc76C-RNAi* wing. (H) Increased width of L5 in *L5-Gal4 UAS-dpp-GFP/+* wing. (I) Partial rescue of L5 width increase by knockdown of Gyc76C in *L5-Gal4 UAS-dpp-GFP/ UAS-gyc76C-RNAi* wing. (J) Comparison of L5 widths between *L5-Gal4*, *L5-Gal4 UAS-dpp-GFP and L5-Gal4 UAS-dpp-GFP/UAS-gyc76C-RNAi* wings. n = 10 for all experimental groups in E and J. * = p<0.001. *L5-Gal4 UAS-gyc76C-RNAi* was not significantly different from *L5-Gal4* (Relative L5 width = 1.04 and 1.08, respectively).
(PDF)

S8 Fig Effects of additional *gyc76C* and *for* mutant clones on PCV development. (A-B’) Anti-pMad staining (red) in homozygous *gyc76C* ^*3L043*^ clones in 28 hour AP *hs-FLP/+; gyc76C* ^*3L043*^ *FRT* ^*2A*^ */hs-GFP RpS17* ^*4*^ *FRT* ^*2A*^ wings. (A,A’) Large clones overlapping the PCV on both surfaces, indicated by the absence of GFP (A, green) resulting in a complete loss of pMad from the PCV region (A’). (B-B’) Smaller clones, indicated by the absence of GFP (green) on the dorsal and ventral epithelium of two individual pupal wings. pMad often persists within homozygous *gyc76C* ^*3L043*^ clones encompassing the parts of the PCV, even when clones overlap on the dorsal and ventral epithelia (arrows). (C,C’) Anti-pMad staining (red) in homozygous *gyc76C* ^*KG0372ex33*^ clones in 28 hour AP *hs-FLP/+; gyc76C* ^*KG0372ex33*^ *FRT* ^*2A*^ */hs-GFP RpS17* ^*4*^ *FRT* ^*2A*^ wings. Large clones overlap on the dorsal and ventral surfaces, resulting in the loss of most, but not all (arrows), pMad from the PCV. (D,E) Anti-DSRF staining (red, white) in homozygous *for* ^*02*^ clones in *hsFlp*; *for* ^*02*^ *FRT* ^40A^/*ubi-RFP FRT* ^*40A*^ 28 hour AP wings. (D) DSRF is still decreased in clone (arrow) on the PCV. (E) Increased DSRF increased in part of PCV adjacent to *for* clone (arrow).
(PDF)

S9 Fig More details on ECM changes. (A-E’). *hh-Gal4 UAS-gyc76C-RNAi* wings containing either *trol-GFP* (A,C-E) or *UAS-rab9*.*YFP* (B). (A,A’) Broad Trol-GFP (green) in L5 and pockets in PCV area extend outside vein regions defined by reduction in DSRF (blue) or heightened pMad (red). (B,B’) High magnification detail showing that anti-Trol staining does not significantly overlap Rab9.YFP vesicles. (C-D’) High magnification details showing Trol-GFP-containing vesicles (green) do not significantly overlap anti-Rab5 (C,C’) or anti-Rab11 (D,D’) staining (purple, white). (E,E’) Moderate magnification detail showing only rare overlap (arrow) between Trol-GFP-containing vesicles (green) and anti-Rab7 staining (red, white). Anti-FasIII staining (blue) shows cell outlines. (F) Apical focus on intervein region of *trol-GFP*; *for* ^*02*^ */ for* ^*02*^ wing showing accumulation of Trol-GFP vesicles. (G-J) Comparison of 6G7 anti-CgIV and anti-Trol staining in *for* ^*02*^ / *CyO* (G,G’,I,I’) and *for* ^*02*^ */ for* ^*02*^ (H,H’,J,J’) wings at 24 (G-H’) and 27–28 (I-J’) hours AP. Diffuse, broad vein accumulation of CgIV and Trol, and alterations in intervein cells, become increasingly apparent from 24 to 27 hours AP, but loss from intervein pockets is more apparent at later stages (see 28–30 hour AP wing in Fig 5B). (K-K’) 6G7 anti-VkgIV and anti-Mmp1 staining in intervein pockets is largely normal in small homozygous *for* ^*02*^ clones, even in outlined region where clones overlap on both dorsal and ventral wing surfaces. (L,M) No defects in 6G7 anti-CgIV staining are apparent in *cv* ^*43*^ (L) or *cv-c* ^*1*^ (M) wings. (N) No defects in anti-Trol staining are apparent in *en-Gal4 UAS-dys-RNAi* wings. (O,P) 28 hour AP *hh-Gal4 UAS-Mmp2-RNAi* wings have slight posterior increases in CgIV aggregates. (Q,R) No obvious difference in anti-βGal staining between *Mmp1-lacZ* (P) and *Mmp1-LacZ hh-Gal4 UAS-gyc76C-RNAi* (Q) wings, except for those that correlate with altered venation. (S-U) Accumulation of Mmp1 in posterior intervein pockets in *hh-Gal4 UAS-gyc76C-RNAi* wings at 26 hours AP (S) and 28–30 hours AP (T), but loss at 32 hours AP (U). (V) Loss of Mmp1 from intervein pockets, but diffuse increase in veins, in 32 hour AP *for* ^*02*^ homozygote. (W,X) Anti-Mmp1 staining in control wild type wings at 26 (W) and 30 (X) hours AP. (Y,Z) DIC optic detail of region posterior to L5 in *hh-Gal4 UAS-gyc76C-RNAi* wings at 30 hours AP, without (Y) and with (Z) *UAS-Mmp2-RNAi*. The abnormally large vesicles normally seen after *gyc76C* knockdown (Y) are much less frequent after knockdown of *Mmp2* (Z).
(PDF)

S10 Fig Candidate proteins unaffected by posterior knockdown of *gyc76*C. Candidates (white, purple) do not change, except in regions of altered venation. All show *hh-Gal4 UAS-gyc76C-RNAi*, except (H,H’) which show wild type control. In some, limits of posterior knockdown is marked with *UAS-GFP* (green), while in others it is shown by approximate position just anterior to L4 (lines). High magnification figures are 2.4x low magnification figures. (A-E) Focus on basal region of maximal integrin concentration, stained with anti-Mys (A-B), anti-Mew (C-C”) or anti-If (D-E). (F,F’) Apico-lateral (nuclear) focus on anti-DSRF and anti-Dei. (G,G’) Basolateral focus and cross-section of anti-Dlp. (H,H’) Basolateral focus and cross-section of anti-Dlp in wild type control. (I) Basal focus on anti-Dg. (J.J’) Basolateral focus on anti-Notch. (K,K’,L) Basolateral focus on anti-Tkv.
(PDF)
