# Supplementary figures and images for: The Gyc76C Receptor Guanylyl Cyclase and the Foraging cGMP-Dependent Kinase Regulate Extracellular Matrix Organization and BMP Signaling in the Developing Wing of Drosophila melanogaster
Source: PLoS Genet. 2015 Oct 6;11(10):e1005576. doi: 10.1371/journal.pgen.1005576 (PMC4595086; doi:10.1371/journal.pgen.1005576)

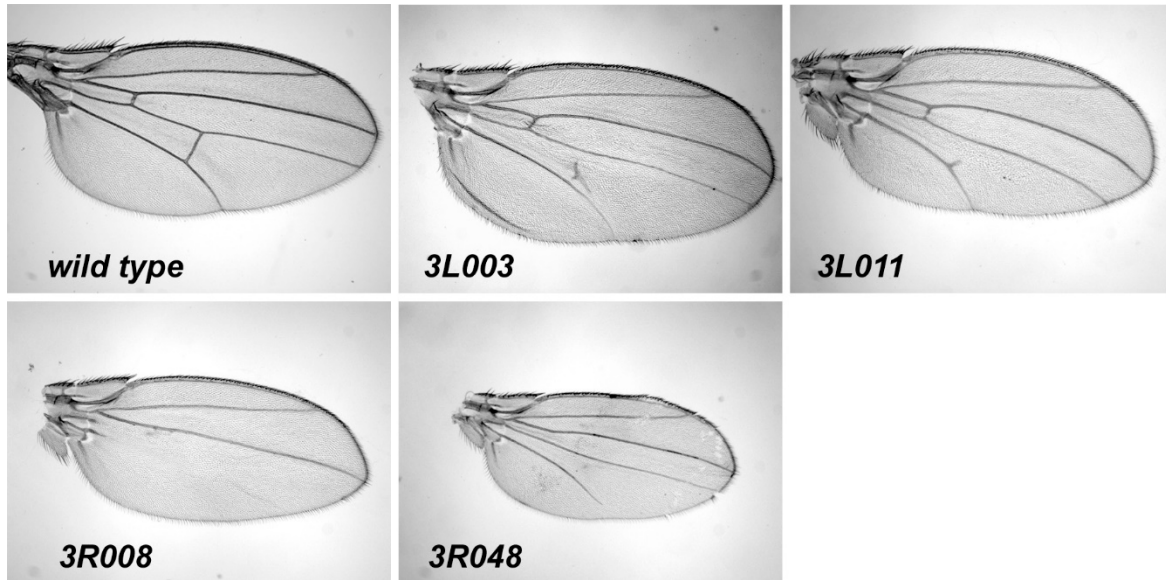

**S1 Fig. Additional venation mutations.**

Supplement: S1 Fig — Adult phenotypes caused by large homozygous posterior clones generated using en-Gal4 UAS-Flp and the Minute method. (PDF) [file pgen.1005576.s001.pdf]

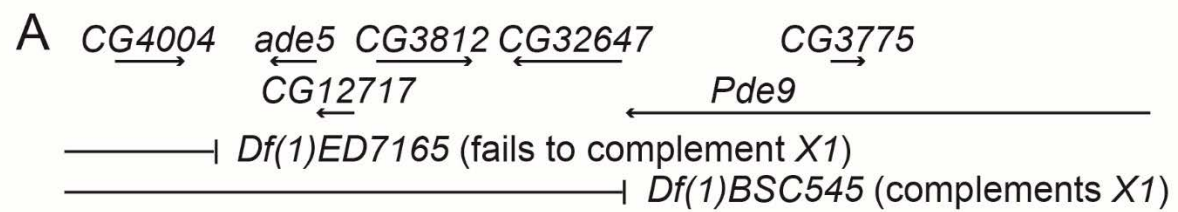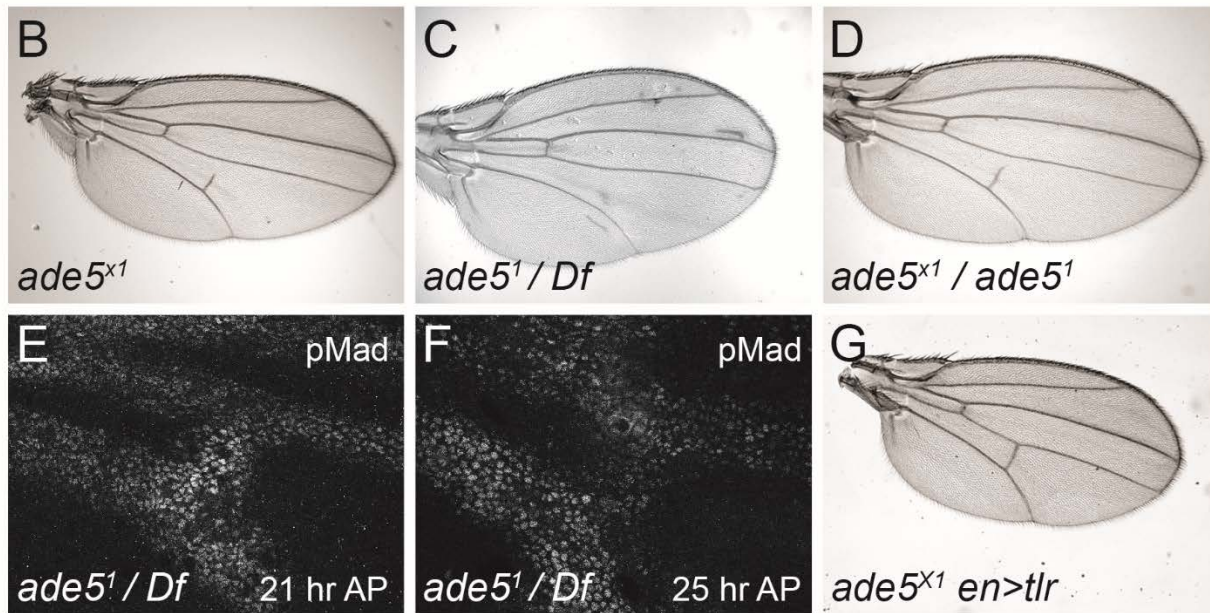

**S3 Fig. *ade5* mutants disrupt PCV development.**

Supplement: S3 Fig — (A) Mapping the X1 mutation to the ade5 region. X1 does not lie at the other end of the deficiencies, since the proximal end of the BSC545 deficiency extends further than the proximal end of ED7165. (B) PCV disruption in ade X1 wing. (C) PCV loss in ade5 1 wing. (D) PCV disruption in ade5 X1/ade5 1 wing. (E,F) anti-pMad staining is present in the PCV region of ade5 1 wings at 21 hours AP (E) but is largely lost by 25 hours AP (F). (G) en-Gal4-driven expression of UAS-tlr rescues the PCV in an ade5 X1 /Y wing. (PDF) [file pgen.1005576.s003.pdf]

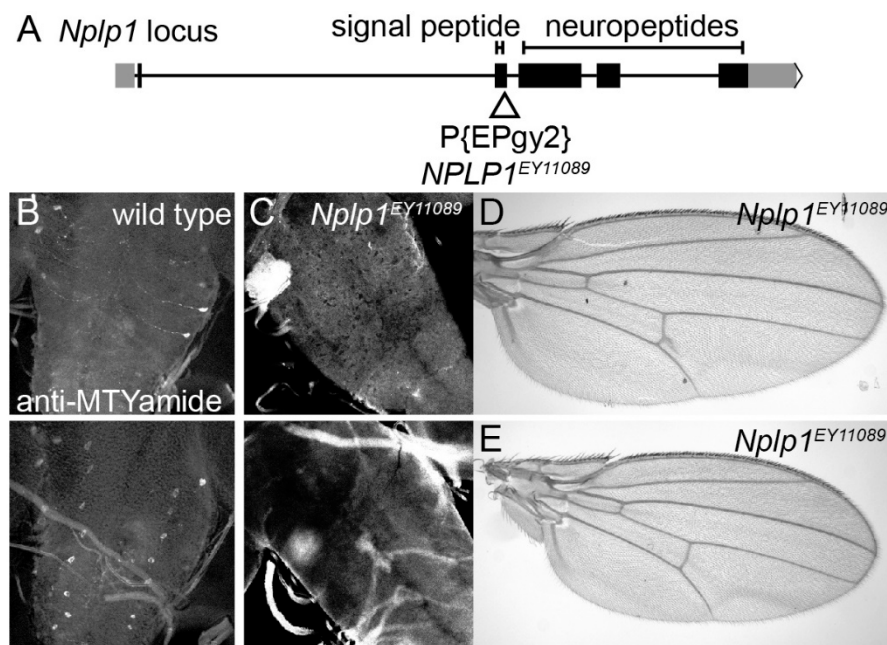

**S4 Fig. *Nplp1* peptides are not required for full Gyc76C activity.**

Supplement: S4 Fig — (A) Nplp1 locus. The P element insertion Nplp1 EY11089 lies in the second coding exon of Nplp1, placing stop codons between the region coding the N-terminal secretion signal peptide and the region coding neuropeptide precursors. (B,C) Larval CNS stained with antiserum against the Nplp1 peptide MTYamide. The strong staining in dorsal and ventral segmentally repeated neuronal cell bodies and axons in wild type (B) is missing in an Nplp1 EY11089 homozygote (C). (D,E) Two adult wings homozygous for Nplp1 EY11089. Unlike after gyc76C knockdown, the PCVs are present and complete, and occasionally show a small ectopic branch (D). (PDF) [file pgen.1005576.s004.pdf]

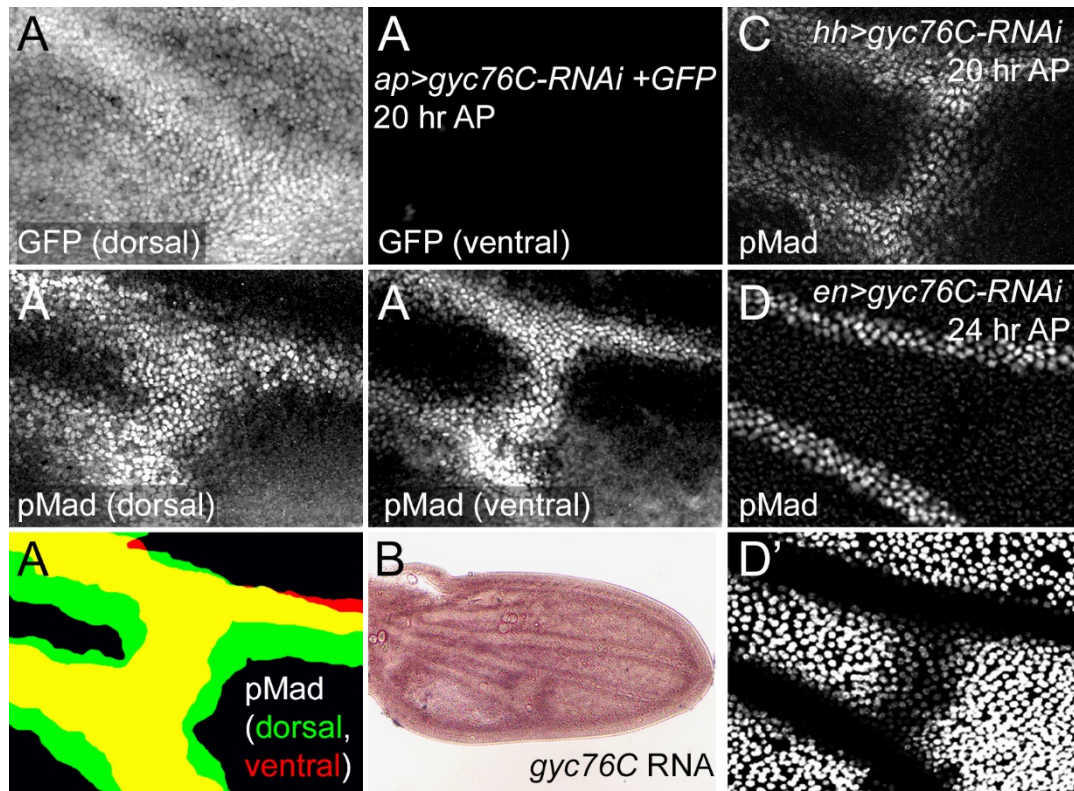

**S5 Fig. More Gyc76C knockdown effects on pupal venation**

Supplement: S5 Fig — (A) Effects of dorsal gyc76C knockdown in 20 hour AP ap-Gal4 UAS-GFP UAS-gyc76C-RNAi wing. Top panels show that ap-driven GFP expression is limited to the dorsal epithelium. Middle panels show that anti-pMad staining is broader in the dorsal epithelium, both around the PCV and the LVs. Lower left panel shows overlay of dorsal (green) and ventral (red) pMad, with overlap in yellow. (B) In situ hybridization to 24 hour AP wing with gyc76C antisense probe. (C) Retention of anti-pMad in PCV of hh-Gal4 UAS-gyc76C-RNAi wing at 20 hours AP. (D,D’) 24 hour AP en-Gal4 UAS-gyc76C-RNAi wings showing loss of pMad (D) but suppression of DSRF (D’) in PCV. (PDF) [file pgen.1005576.s005.pdf]

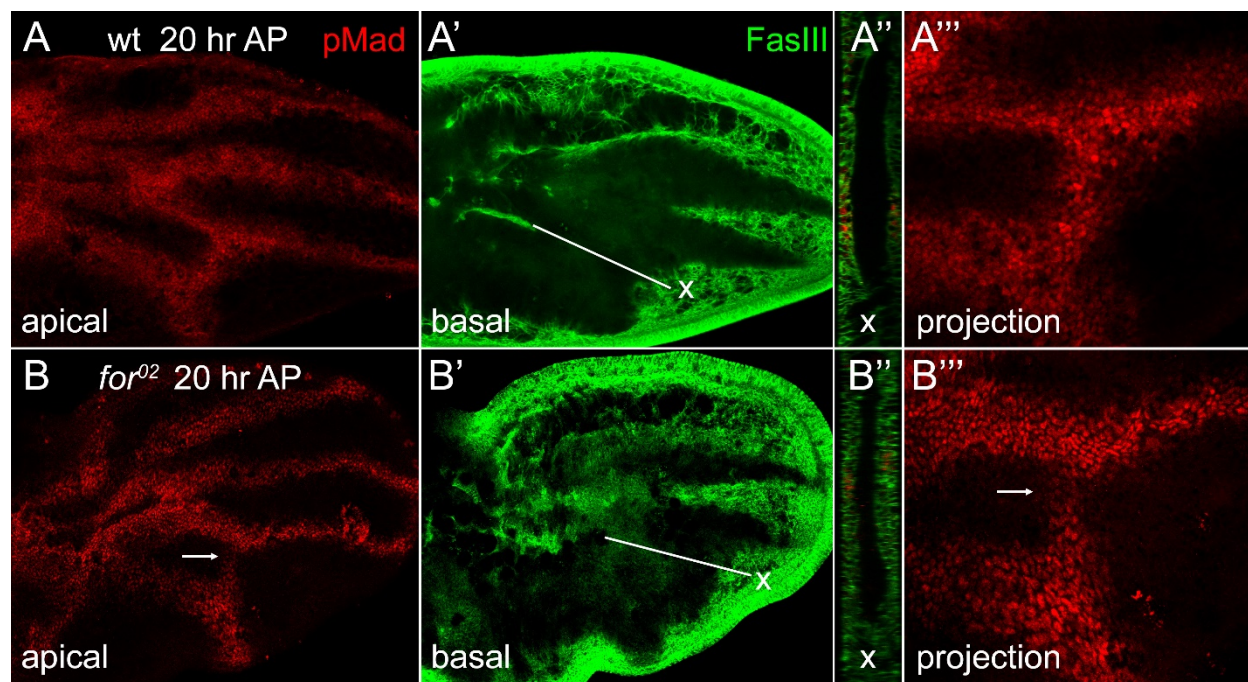

**S6 Fig. Early disruption of PCV in *for* mutant.**

Supplement: S6 Fig — Comparison of wild type (A-A”‘) and for 02 homozygous (B-B”‘) pupal wings at 20 hours AP, with anti-pMad staining in red and anti-FasIII-stained cell membranes in green. (A,B) Low magnification image of single apical (nuclear) focal plane showing normal (A) and partially disrupted (B, arrow) pMad in PCV. (A’,B’) Low magnification image of basal focal plane, showing green membranes where dorsal and ventral epithelia have attached, and the dark basal lumen where epithelia have not yet attached. The lumen does not yet define physical veins in the region of the PCV. (A”,B”) Cross-sections (x) reconstructed from high magnification z-series images, along x lines in A’ or B’. Widths of normal (A”) or abnormal (B”) PCV pMad (red) regions are much narrower than the basal lumens. (A”‘,B”‘) High magnification projections of z-series images from all the pMad-containing focal planes on one epithelium, again showing PCV disruption (arrow) in the for mutant. (PDF) [file pgen.1005576.s006.pdf]

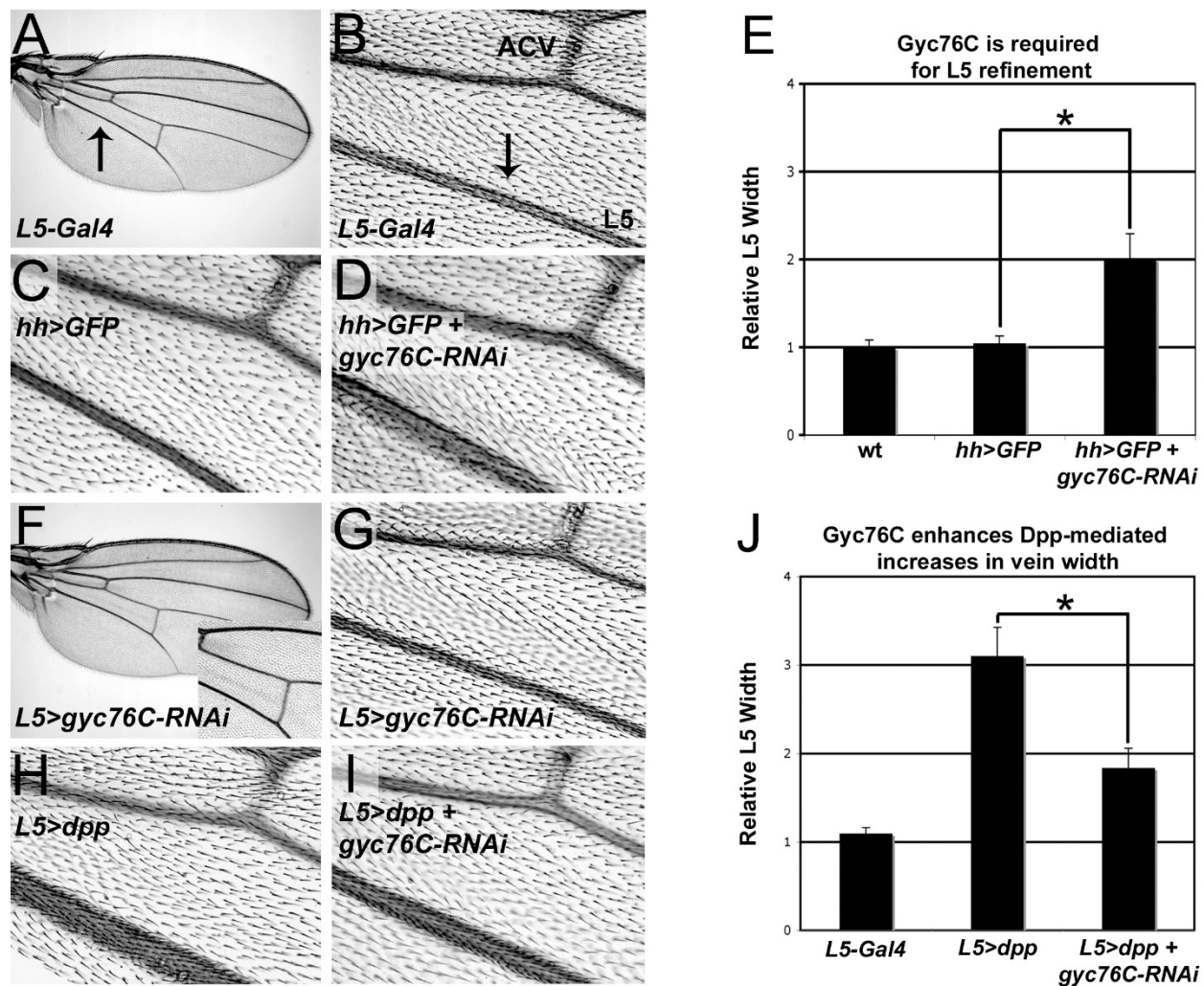

**S7 Fig. Effects of Gyc76C on adult vein width.**

Supplement: S7 Fig — (A,B) L5-Gal4 (control) adult wing. Arrows demarcate the region of L5 posterior to the ACV, used in measurements of vein width. (C) L5 width in hh-Gal4 UAS-GFP/+ flies is similar to wild-type and L5-Gal4. (D) Increased width of L5 in hh-Gal4 UAS-GFP/ UAS-gyc76C-RNAi (VDRC 6552) flies. (E) Comparison of L5 widths between wild type (wt), hh-Gal4 UAS-GFP/+ and hh-Gal4 UAS-GFP/UAS-gyc76C-RNAi wings. (F and G) Normal L5 width in L5-Gal4 UAS-gyc76C-RNAi wing. (H) Increased width of L5 in L5-Gal4 UAS-dpp-GFP/+ wing. (I) Partial rescue of L5 width increase by knockdown of Gyc76C in L5-Gal4 UAS-dpp-GFP/ UAS-gyc76C-RNAi wing. (J) Comparison of L5 widths between L5-Gal4, L5-Gal4 UAS-dpp-GFP and L5-Gal4 UAS-dpp-GFP/UAS-gyc76C-RNAi wings. n = 10 for all experimental groups in E and J. * = p<0.001. L5-Gal4 UAS-gyc76C-RNAi was not significantly different from L5-Gal4 (Relative L5 width = 1.04 and 1.08, respectively). (PDF) [file pgen.1005576.s007.pdf]

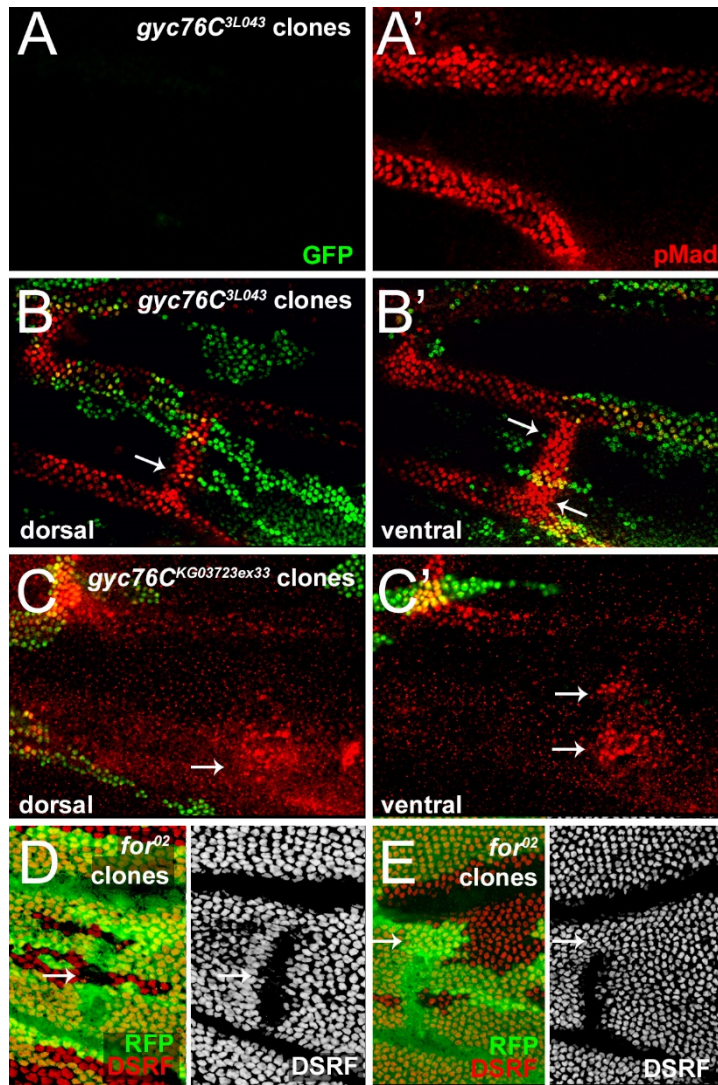

**S8 Fig. Effects of additional *gyc76C* and *for* mutant clones on PCV development.**

Supplement: S8 Fig — (A-B’) Anti-pMad staining (red) in homozygous gyc76C 3L043 clones in 28 hour AP hs-FLP/+; gyc76C 3L043 FRT 2A /hs-GFP RpS17 4 FRT 2A wings. (A,A’) Large clones overlapping the PCV on both surfaces, indicated by the absence of GFP (A, green) resulting in a complete loss of pMad from the PCV region (A’). (B-B’) Smaller clones, indicated by the absence of GFP (green) on the dorsal and ventral epithelium of two individual pupal wings. pMad often persists within homozygous gyc76C 3L043 clones encompassing the parts of the PCV, even when clones overlap on the dorsal and ventral epithelia (arrows). (C,C’) Anti-pMad staining (red) in homozygous gyc76C KG0372ex33 clones in 28 hour AP hs-FLP/+; gyc76C KG0372ex33 FRT 2A /hs-GFP RpS17 4 FRT 2A wings. Large clones overlap on the dorsal and ventral surfaces, resulting in the loss of most, but not all (arrows), pMad from the PCV. (D,E) Anti-DSRF staining (red, white) in homozygous for 02 clones in hsFlp; for 02 FRT 40A/ubi-RFP FRT 40A 28 hour AP wings. (D) DSRF is still decreased in clone (arrow) on the PCV. (E) Increased DSRF increased in part of PCV adjacent to for clone (arrow). (PDF) [file pgen.1005576.s008.pdf]

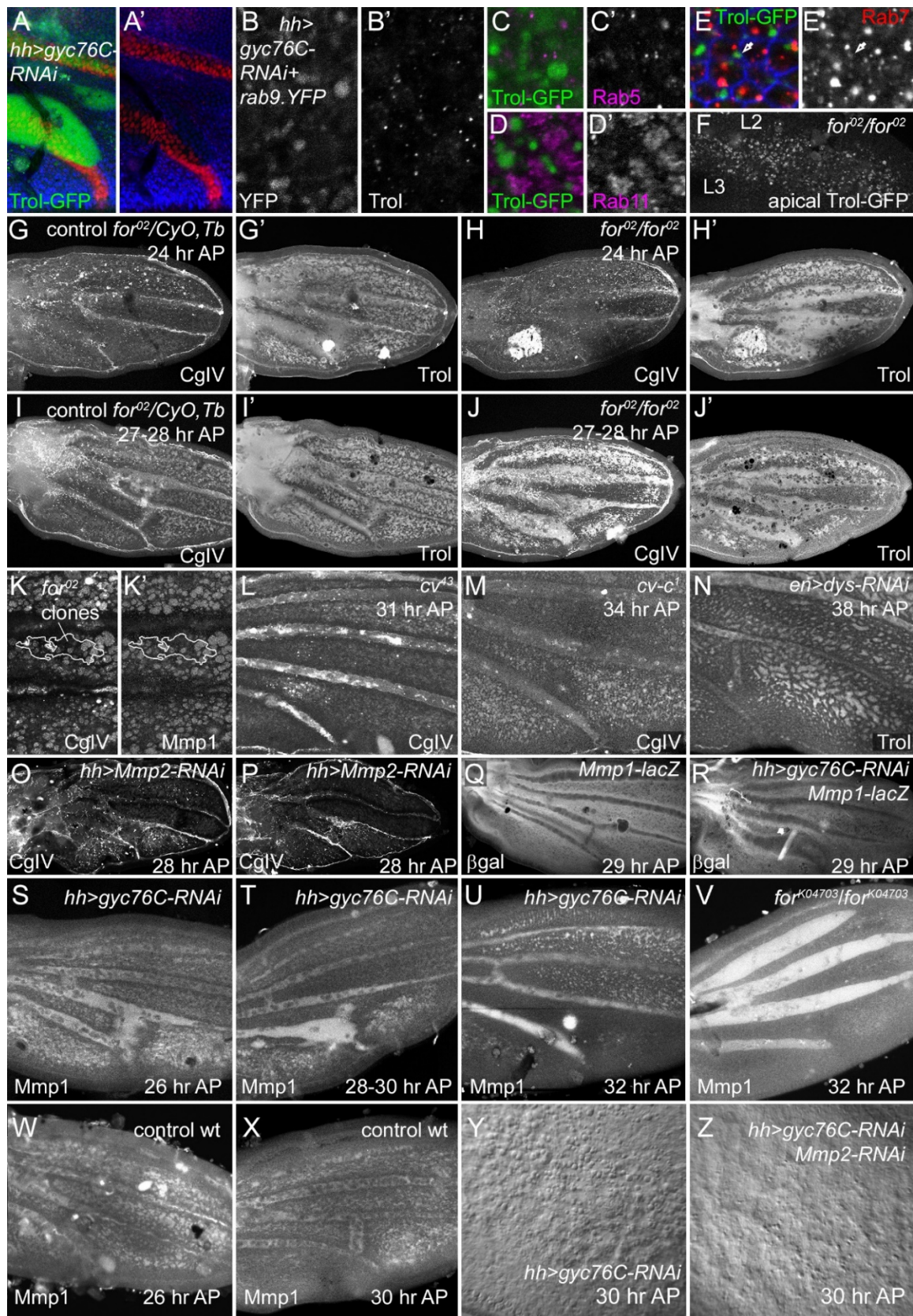

**S9 Fig. More details on ECM changes.**

Supplement: S9 Fig — (A-E’). hh-Gal4 UAS-gyc76C-RNAi wings containing either trol-GFP (A,C-E) or UAS-rab9.YFP (B). (A,A’) Broad Trol-GFP (green) in L5 and pockets in PCV area extend outside vein regions defined by reduction in DSRF (blue) or heightened pMad (red). (B,B’) High magnification detail showing that anti-Trol staining does not significantly overlap Rab9.YFP vesicles. (C-D’) High magnification details showing Trol-GFP-containing vesicles (green) do not significantly overlap anti-Rab5 (C,C’) or anti-Rab11 (D,D’) staining (purple, white). (E,E’) Moderate magnification detail showing only rare overlap (arrow) between Trol-GFP-containing vesicles (green) and anti-Rab7 staining (red, white). Anti-FasIII staining (blue) shows cell outlines. (F) Apical focus on intervein region of trol-GFP; for 02 / for 02 wing showing accumulation of Trol-GFP vesicles. (G-J) Comparison of 6G7 anti-CgIV and anti-Trol staining in for 02 / CyO (G,G’,I,I’) and for 02 / for 02 (H,H’,J,J’) wings at 24 (G-H’) and 27–28 (I-J’) hours AP. Diffuse, broad vein accumulation of CgIV and Trol, and alterations in intervein cells, become increasingly apparent from 24 to 27 hours AP, but loss from intervein pockets is more apparent at later stages (see 28–30 hour AP wing in Fig 5B). (K-K’) 6G7 anti-VkgIV and anti-Mmp1 staining in intervein pockets is largely normal in small homozygous for 02 clones, even in outlined region where clones overlap on both dorsal and ventral wing surfaces. (L,M) No defects in 6G7 anti-CgIV staining are apparent in cv 43 (L) or cv-c 1 (M) wings. (N) No defects in anti-Trol staining are apparent in en-Gal4 UAS-dys-RNAi wings. (O,P) 28 hour AP hh-Gal4 UAS-Mmp2-RNAi wings have slight posterior increases in CgIV aggregates. (Q,R) No obvious difference in anti-βGal staining between Mmp1-lacZ (P) and Mmp1-LacZ hh-Gal4 UAS-gyc76C-RNAi (Q) wings, except for those that correlate with altered venation. (S-U) Accumulation of Mmp1 in posterior intervein pockets in hh-Gal4 UAS-gyc76C-RNAi wings at 26 ho [file pgen.1005576.s009.pdf]

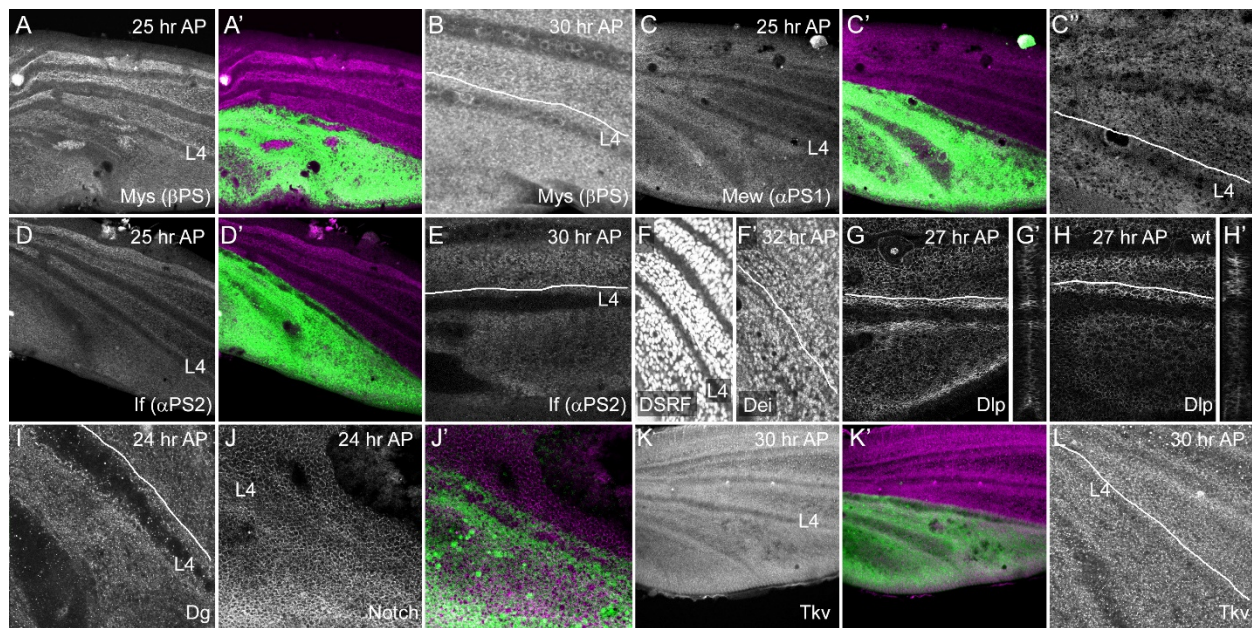

**S10 Fig. Candidate proteins unaffected by posterior knockdown of *gyc76C*.**

Supplement: S10 Fig — Candidates (white, purple) do not change, except in regions of altered venation. All show hh-Gal4 UAS-gyc76C-RNAi, except (H,H’) which show wild type control. In some, limits of posterior knockdown is marked with UAS-GFP (green), while in others it is shown by approximate position just anterior to L4 (lines). High magnification figures are 2.4x low magnification figures. (A-E) Focus on basal region of maximal integrin concentration, stained with anti-Mys (A-B), anti-Mew (C-C”) or anti-If (D-E). (F,F’) Apico-lateral (nuclear) focus on anti-DSRF and anti-Dei. (G,G’) Basolateral focus and cross-section of anti-Dlp. (H,H’) Basolateral focus and cross-section of anti-Dlp in wild type control. (I) Basal focus on anti-Dg. (J.J’) Basolateral focus on anti-Notch. (K,K’,L) Basolateral focus on anti-Tkv. (PDF) [file pgen.1005576.s010.pdf]
