# Supplementary material for: The Gyc76C Receptor Guanylyl Cyclase and the Foraging cGMP-Dependent Kinase Regulate Extracellular Matrix Organization and BMP Signaling in the Developing Wing of Drosophila melanogaster
Source: PLoS Genet. 2015 Oct 6;11(10):e1005576. doi: 10.1371/journal.pgen.1005576 (PMC4595086; doi:10.1371/journal.pgen.1005576)
Supplement: S2 Fig — Predicted duplicate gene names have the same color. The duplication has only been observed in a subset of the iso-1 strain used for BDGP sequencing, and was likely caused by the mobilization of genomic DNA around an original Doc insertion [57]. When present, the aberration duplicates CG14101 (termed CG42529 in one duplicate) and the coding exons, but not promoter or 5’ UTR exons, of gyc76C (termed GC42637 in one duplicate, although it is not certain that the longer GC42637 primary transcript is made). The duplication is very unlikely to be present in the 3L043 chromosome: the duplication is not even present in all iso-1 flies, and Canton-S and Oregon-R wild type strains lack both the Doc insertions and the duplication [57]. Since the gyc76C L635H mutation likely causes the 3L043 genotype, it is doubtful that the 3L043 chromosome contains a second, functioning duplication of gyc76C coding exons. (PDF) [file pgen.1005576.s002.pdf]

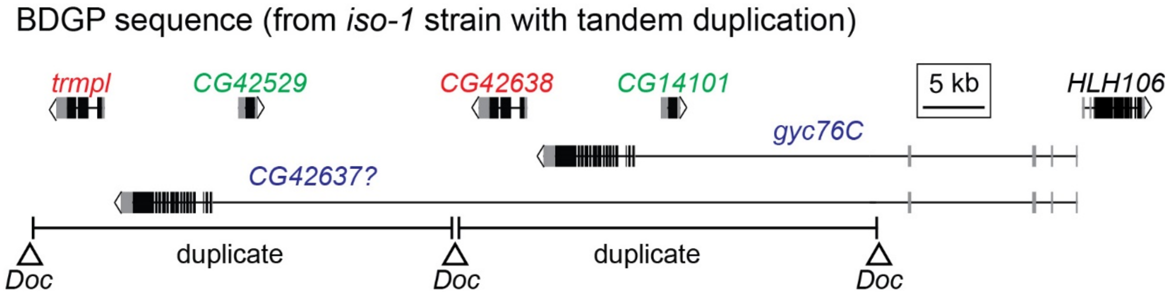

S2 Fig. Tandem duplication of *gyc76C* genomic region in BDGP sequence.
